# Promoted hydrogenation of CO_2_ to methanol over single-atom Cu sites with Na^+^-decorated microenvironment

**DOI:** 10.1093/nsr/nwae114

**Published:** 2024-03-22

**Authors:** Li-Li Ling, Xinyu Guan, Xiaoshuo Liu, Xiao-Mei Lei, Zhongyuan Lin, Hai-Long Jiang

**Affiliations:** Hefei National Research Center for Physical Sciences at the Microscale, Department of Chemistry, University of Science and Technology of China, Hefei 230026, China; Hefei National Research Center for Physical Sciences at the Microscale, Department of Chemistry, University of Science and Technology of China, Hefei 230026, China; School of Energy and Power Engineering, North China Electric Power University, Baoding 071003, China; School of Energy and Environment, Southeast University, Nanjing 210096, China; Hefei National Research Center for Physical Sciences at the Microscale, Department of Chemistry, University of Science and Technology of China, Hefei 230026, China; Hefei National Research Center for Physical Sciences at the Microscale, Department of Chemistry, University of Science and Technology of China, Hefei 230026, China; Hefei National Research Center for Physical Sciences at the Microscale, Department of Chemistry, University of Science and Technology of China, Hefei 230026, China

**Keywords:** single-atom sites, metal–organic framework, CO_2_ hydrogenation, heterogeneous catalysis, alkali-metal decoration

## Abstract

Although single-atom Cu sites exhibit high efficiency in CO_2_ hydrogenation to methanol, they are prone to forming Cu nanoparticles due to reduction and aggregation under reaction conditions, especially at high temperatures. Herein, single-atom Cu sites stabilized by adjacent Na^+^ ions have been successfully constructed within a metal–organic framework (MOF)-based catalyst, namely MOF-808-NaCu. It is found that the electrostatic interaction between the Na^+^ and H^δ−^ species plays a pivotal role in upholding the atomic dispersion of Cu in MOF-808-NaCu during CO_2_ hydrogenation, even at temperatures of up to 275°C. This exceptional stabilization effect endows the catalyst with excellent activity (306 g·kg_cat_^−1^·h^−1^), high selectivity to methanol (93%) and long-term stability at elevated reaction temperatures, far surpassing the counterpart in the absence of Na^+^ (denoted as MOF-808-Cu). This work develops an effective strategy for the fabrication of stable single-atom sites for advanced catalysis by creating an alkali-decorated microenvironment in close proximity.

## Teaser

Single-atom Cu sites are stabilized by Na+ decorated microenvironment in a metal-organic framework, offering high activity and CH3OH selectivity in the hydrogenation of carbon dioxide up to 275°C.

## INTRODUCTION

Carbon dioxide (CO_2_), one of the prominent contributors to the greenhouse effect, has generated significant environmental and climate concerns in recent years. In response to this issue, the direct conversion of CO_2_ into chemicals and/or fuels through hydrogenation is widely acknowledged as a viable strategy for reducing both CO_2_ emissions and fossil fuel consumption [1–5]. Although copper (Cu)-based catalysts, such as Cu/ZnO/Al_2_O_3_, have been successfully adopted for CO_2_ hydrogenation in industrial applications, they face considerable difficulty in simultaneously meeting the demands of high CO_2_ conversion and methanol selectivity under demanding operating conditions [6–9]. It is noteworthy that the conversion of CO_2_ and selectivity to methanol are highly dependent on the nature of surface Cu species. Specifically, methanol production is favored at the interfaces between the electron-deficient Cu sites (Cu^δ+^) and supporting materials [10], whereas the reverse water gas shift coupled with carbon monoxide hydrogenation (RWGS + CO-hydro) pathway is promoted on the naked surface of Cu nanoparticles (NPs), leading to associated production of CO [11]. In fact, though the reaction kinetics is accelerated at elevated reaction temperatures, Cu NPs display even lower selectivity to methanol due to the endothermic nature of the RWGS step and the exothermic nature of CO hydrogenation, usually giving rise to the trade-off between activity and selectivity [1,2,9]. Therefore, it is highly desired for the construction and stabilization of abundant Cu^δ+^–support interfaces to promote efficient CO_2_ hydrogenation with high methanol selectivity.

In this context, Cu single-atom catalysts (SACs) have demonstrated notable promise in achieving exceptional selectivity to methanol [12] on account of their exceptional atomic utilization efficiency and abundant metal–support interfaces [13–15]. Of particular note is the attachment of single-atom metal sites to metal oxides [16–18], which has attracted significant attention in heterogeneous catalytic hydrogenation, benefitting from the improved dissociation of hydrogen aided by adjacent oxygen atoms [19,20]. Unfortunately, Cu SACs often suffer from challenges such as reduction and aggregation, especially in an hydrogenated atmosphere and at elevated temperatures, leading to the generation of Cu NPs and reduction of methanol selectivity [11]. Consequently, the stabilization of single-atom Cu sites remains a formidable obstacle in high-performance CO_2_ hydrogenation.

Recently, it was reported that isolated nanoglue islands located on high-surface-area supports are able to concurrently improve the stability and reactivity of confined atomically dispersed metal sites [21–23]. Metal–organic frameworks (MOFs)—a class of crystalline porous materials assembled by metal ions/clusters and organic linkers—have emerged as ideal platforms for immobilizing metal sites and heterogeneous catalysis [24–30]. Particularly, the periodically separated metal–oxo clusters in MOFs featuring abundant oxo/hydroxo groups can serve as excellent binding sites for supporting single metal sites, effectively mimicking isolated metal oxide islands as highly active supports [22,31–33]. It is worth noting that, despite methanol production by CO_2_ hydrogenation over MOF-based materials having been reported [34–40], the only study on MOF-embedded atomically dispersed Cu sites has thus far been limited to 100°C [41]. So far, the stability and performance of MOF-based Cu SACs at moderately high operating temperatures (typically >200°C), which are important for improving the reaction kinetics of CO_2_ hydrogenation, have yet to be explored.

**Figure 1.**
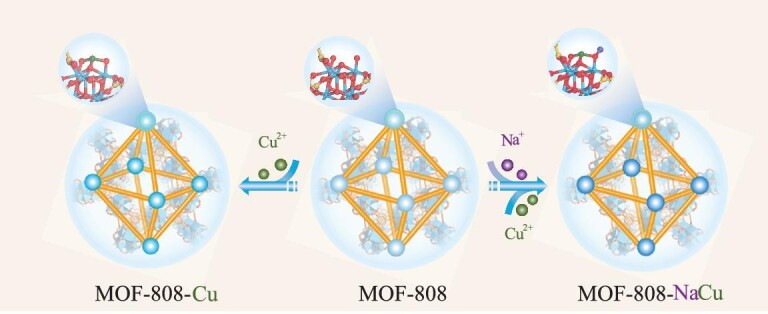
Illustration showing the construction of single-atom Cu sites in the presence or absence of an Na^+^-decorated microenvironment based on MOF-808.

Herein, MOF-808, a Zr-MOF constructed with Zr–oxo clusters featuring abundant –O/OH*_x_* groups, is adopted to bind single-atom Cu sites with neighboring Na^+^ ions, affording MOF-808-NaCu (Fig. 1), for CO_2_ hydrogenation. Remarkably, the unique microenvironment of Cu sites created by the adjacent Na^+^ ions plays a crucial role in stabilizing the single-atom Cu sites. Consequently, MOF-808-NaCu displays exceptional methanol selectivity exceeding 93% at temperatures ranging from 150°C to even 275°C, with the maximum space time yield (STY) of methanol reaching 306 g⋅kg_cat_^−1^⋅h^−1^ at 275°C. In stark contrast, MOF-808-Cu, the counterpart in the absence of Na^+^ decoration, produces a significantly lower STY of 28 g⋅kg_cat_^−1^⋅h^−1^ and selectivity of 35% under similar conditions.

## RESULTS AND DISCUSSION

**Figure 2.**
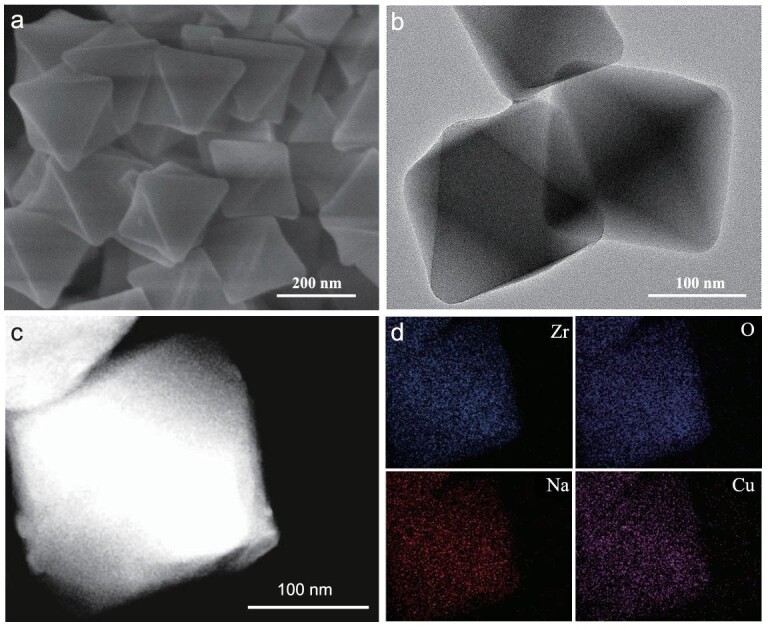
SEM, TEM and HAADF–STEM images of MOF-808-NaCu. (a) SEM, (b) TEM images, (c) HAADF–STEM of MOF-808-NaCu and (d) the corresponding Zr, O, Na and Cu elemental mapping images.

The synthesis of MOF-808-NaCu follows a two-step metalation procedure (Fig. 1). Firstly, pristine MOF-808 was immersed in a solution of sodium tert-butoxide in tetrahydrofuran (THF) to give Na^+^-decorated MOF-808-Na. Subsequently, a facile post-synthetic metal substitution strategy was employed to introduce single-atom Cu sites, involving the reaction with Cu(CH_3_CN)_4_BF_4_ in THF (see supporting information, Experimental Section). As a control, MOF-808-Cu was also prepared through a similar reaction involving MOF-808 and Cu(CH_3_CN)_4_BF_4_ in THF. As revealed by powder X-ray diffraction patterns, all these modified MOFs demonstrate crystalline structures similar to that of the pristine MOF-808, with no observed Cu-related inorganic crystalline phases (Fig. S1). Nitrogen sorption isotherms at 77 K indicate the inherited microporous architectures of MOF-808 after Cu/Na decoration, albeit with partially decreased surface areas (Fig. S2). Thermogravimetric analysis (TGA) under nitrogen demonstrates similar stability for MOF-808, MOF-808-Cu and MOF-808-NaCu (Figs S3–S5). Scanning electron microscopy (SEM) and transmission electron microscopy (TEM) observation indicate similar octahedral morphologies of MOF-808-NaCu and MOF-808-Cu, with sizes of ∼200 nm, and no visible Cu NPs can be observed in the TEM images (Fig. 2a and b, and Fig. S6). A more detailed investigation of these nanocrystals using high-angle annular dark-field scanning transmission electron microscopy (HAADF–STEM) in conjunction with energy-dispersive X-ray mapping further verifies the uniform distribution of Na and Cu elements throughout the MOF particle (Fig. 2c and 2d). The successful implantation of Na and Cu can be further supported by using inductively coupled plasma optical emission spectrometry (ICP–OES) (Table S1).

Additionally, the binding sites of Na and Cu within MOF-808 are elucidated using diffuse reflection infrared Fourier transform spectroscopy (DRIFTS). Compared with the pristine MOF-808, noticeable reduction in the –OH/OH_2_ adsorption peak (at ∼3670 cm^−1^) can be observed upon metal loading, indicating the interaction between Na^+^/Cu^2+^ and –OH/OH_2_ groups (Fig. S7). Therefore, the binding sites for both Na and Cu species are associated with the abundant –OH/OH_2_ groups situated on the Zr–oxo clusters. Given the nearly unchanged –OH/OH_2_ peak intensity after Cu^2+^ modification onto MOF-808-Na, together with the reduced Na loading amount, the ion exchange process between Cu^2+^ and the initially anchored Na^+^ is assumed. These findings collectively imply that the Cu sites are surrounded by a Na^+^-decorated microenvironment in MOF-808-NaCu.

**Figure 3.**
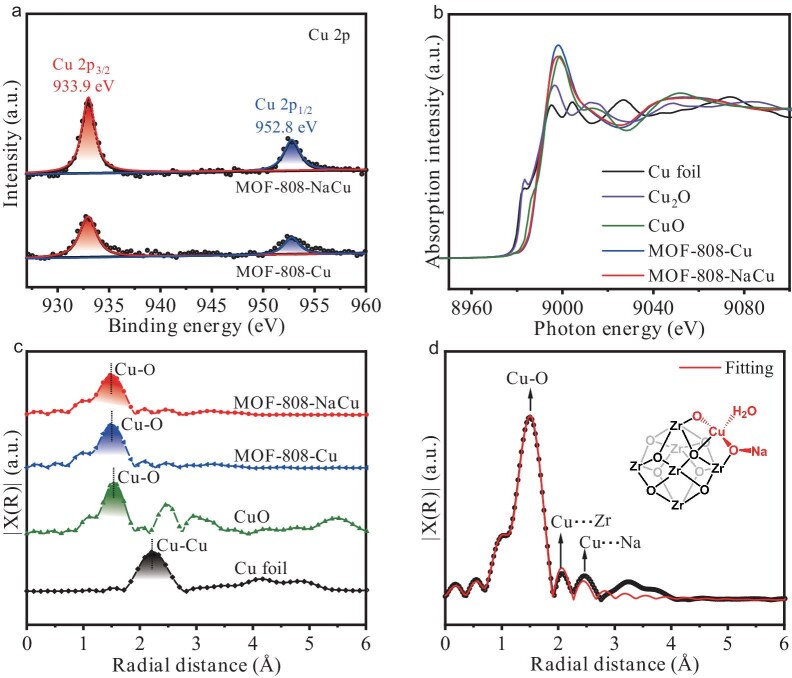
Local structure definition of MOF-808-NaCu. (a) The Cu 2p XPS spectra of MOF-808-NaCu and MOF-808-Cu. (b) The Cu K-edge XANES spectra and (c) FT–EXAFS spectra of MOF-808-NaCu, MOF-808-Cu and related control samples. (d) The EXAFS fitting of MOF-808-NaCu (inset: optimized local structure of Zr–oxo cluster modified with Na^+^ and Cu^2+^).

Subsequently, the chemical and electronic states of Na/Cu are probed. The X-ray photoelectron spectroscopy (XPS) spectra suggest the extra Na 1s peak at 1072 eV for MOF-808-NaCu compared with MOF-808-Cu, indicating the presence of the O–Na bonding in the former (Figs S8 and S9) [42]. The Cu 2p XPS spectra give characteristic signals of Cu species at ∼933.1 and ∼952.8 eV for MOF-808-NaCu, corresponding to the +2 oxide state (Fig. 3a). Similar Cu signals are also observed for MOF-808-Cu, implying that the Cu electronic state is almost not influenced by the Na^+^ decoration (Fig. 3a). Moreover, the Cu K-edge X-ray absorption near-edge structure (XANES) spectra for MOF-808-NaCu and MOF-808-Cu display adsorption edge positions similar to that of CuO, further indicating the presence of Cu species in the +2 oxide state for both materials (Fig. 3b). Fourier transform-extended X-ray absorption fine structure (FT–EXAFS) analysis for both materials reveals a prominent peak corresponding to Cu–O bonding (at ∼1.52 Å), while no peak related to Cu–Cu scattering (∼2.3 Å) is detected, confirming the absence of Cu clusters or NPs (Fig. 3c). To gain deeper insight into the chemical configurations in MOF-808-NaCu, EXAFS fitting has been conducted (Fig. 3d). The fitting result demonstrates that the Cu atom is coordinated by four O atoms in the first shell, with the second shell positioned at 2.12 and 2.33 Å, assignable to Cu–Zr and Cu–Na, respectively (Fig. 3d). In light of these findings, the local structure of Zr–oxo–NaCu clusters should be generated in MOF-808-NaCu (Fig. 3d, inset).

Encouraged by the successful fabrication of MOF-based single-atom Cu sites, the CO_2_ hydrogenation has been conducted across a temperature range of 150–275°C, using a CO_2_/H_2_ gas mixture with a feed ratio of 1/3 at a pressure of 3.5 MPa. The CO_2_ hydrogenation can be promoted over both MOF-808-Cu and MOF-808-NaCu, while no discernible hydrogenation product is detected over the Cu-free MOFs, i.e. MOF-808 or MOF-808-Na (Fig. S10). Furthermore, the selectivity and STY of methanol in MOF-808-NaCu improve along with increased Cu loading from 2 to 4.5 wt% (Fig. S11). The results reflect that the Cu site should be an active center in the reaction, which can be further supported by temperature-programmed desorption tests using CO_2_ as the probing molecule. Notably, the CO_2_ desorption peak displays a noticeable shift to a higher temperature after the introduction of Cu sites (∼305°C for MOF-808-Cu and ∼295°C for MOF-808-NaCu) compared with that of pristine MOF-808 (∼245°C), suggesting Cu as the potential CO_2_ adsorption site (Fig. S12). Additionally, despite the similar CO_2_ desorption temperatures, MOF-808-NaCu displays higher CO_2_ adsorption capacity than MOF-808-Cu (Fig. S12), hinting that CO_2_ adsorption can be promoted by the Na^+^-decorated microenvironment.

**Figure 4.**
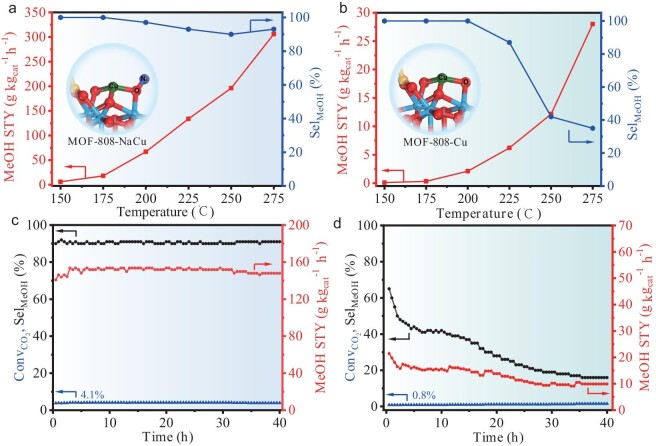
Catalytic performance for CO_2_ hydrogenation. The STY and selectivity of methanol at different temperatures over (a) MOF-808-Cu and (b) MOF-808-NaCu. Reaction conditions: CO_2_/H_2_ volume ratio of 1/3 with reaction pressure of 3.5 MPa and reaction temperatures from 150°C to 275°C. Catalyst stability test for 40 h over (c) MOF-808-NaCu and (d) MOF-808-Cu. Reaction conditions: CO_2_/H_2_ volume ratio of 1/3 with reaction pressure of 3.5 MPa and reaction temperature of 250°C.

For both Cu-based catalysts, the methanol STY gradually increases with the increasing temperature. Remarkably, MOF-808-NaCu achieves the methanol STY peak of 306 g⋅kg_cat_^−1^⋅h^−1^ at 275°C (Fig. 4a), which is 10.9 times higher than that of MOF-808-Cu (28 g⋅kg_cat_^−1^⋅h^−1^) under similar conditions (Fig. 4b), indicating the accelerated CO_2_ hydrogenation with Na^+^ decoration. Although both catalysts exhibit methanol selectivity of >90% at relatively low temperatures (150–200°C), the methanol product gradually shifts towards CO over MOF-808-Cu at elevated reaction temperatures, giving methanol selectivity as low as 35% at 275°C. Strikingly, the high methanol selectivity of >93% can be maintained over MOF-808-NaCu across all evaluated temperatures from 150°C to 275°C, which is apparently different from previously reported Cu SACs that typically possess excellent selectivity at relatively low temperatures only [11,12,41], highlighting the importance of the Na^+^-decorated microenvironment. Furthermore, the physical mixture of MOF-808-Na and MOF-808-Cu (denoted mix-MOF-808-Cu/Na) shows modest methanol STY (36 g⋅kg_cat_^−1^⋅h^−1^) and selectivity (41%) at 275°C (Fig. S10), emphasizing the pivotal role of the close proximity between Na and Cu sites in the methanol production. In addition, the generality of this microenvironment-modulated strategy has been assessed by substituting Na^+^ ions with K^+^ or Cs^+^ ions. Notably, compared with MOF-808-Cu, both the production rate and the selectivity of methanol are significantly improved with MOF-808-KCu and MOF-808-CsCu catalysts, although they are apparently lower than those of MOF-808-NaCu, illustrating the promoting effect of various alkali ions (Figs S10, S13 and S14). Similarly, the physical mixture of MOF-808-Cu and MOF-808-K or MOF-808-Cs (denoted mix-MOF-808-Cu/K or mix-MOF-808-Cu/Cs, respectively) exhibits inferior methanol STY and selectivity compared with their corresponding counterparts with close proximity between alkali-metal and Cu sites (Fig. S10).

Remarkably, the exceptional hydrogenation performance of MOF-808-NaCu can be preserved, even extending the reaction time length to 40 h at 250°C, exhibiting a nearly unaltered CO_2_ conversion (Conv_CO2_) of 4.1%, methanol STY of 148 g⋅kg_cat_^−1^⋅h^−1^ and methanol selectivity (Sel_MeOH_) of 91% (Fig. 4c), indicating its excellent stability during the CO_2_ hydrogenation process. In sharp contrast, the performance of MOF-808-Cu decays rapidly under similar conditions, displaying a methanol STY of only 9.8 g⋅kg_cat_^−1^⋅h^−1^ and selectivity of 16% after a reaction time of 40 h (Fig. 4d). The results underscore the significant effect of Na^+^ in endowing atomically dispersed Cu sites with elevated activity, selectivity and stability. As controls, the commercial ternary Cu-ZnO-Al_2_O_3_ and ZnO-ZrO_2_ catalysts have been evaluated, in which they present their respective drawbacks, in comparison with MOF-808-NaCu, under similar reaction conditions (Figs S15 and S16).

To probe the possible reason behind the significantly different performance between MOF-808-NaCu and MOF-808-Cu, the chemical environment around Cu sites is investigated for both catalysts after reaction (denoted as MOF-808-NaCu-T and MOF-808-Cu-T, respectively, where T represents the catalysis temperature). The crystallinity of MOF-808-NaCu can be almost retained after the catalytic reaction (Fig. S17). The XPS and Cu K-edge XANES spectra of MOF-808-NaCu-275 support that the original +2 oxide state of the Cu species can be preserved even after catalysis at 275°C, as indicated by negligible alterations in the Cu *2p_3/2_* peak (932.9 eV) and the Cu K-edge position (Figs S18 and S19). Furthermore, FT–EXAFS analysis of MOF-808-NaCu-275 highlights Cu–O scattering at ∼1.52 Å without Cu–Cu scattering, manifesting in the atomic dispersion of Cu species after catalysis (Fig. S20). By contrast, although the +2 oxide state of Cu can be retained after catalysis at temperatures of ≤200°C, the Cu^0^ species gradually emerges in MOF-808-Cu as the temperature further increases (Fig. S21). Notably, the majority of Cu(II) species have been reduced to Cu^0^ at 275°C, as proven by the significantly shifted XPS peak of MOF-808-Cu-275, which appears at ∼931.6 eV (Fig. S18). This transformation is further confirmed by its similar absorption edge to that of Cu foil in the Cu K-edge XANES spectra (Fig. S22) and the presence of Cu–Cu scattering at ∼2.21 Å in the FT–EXAFS analysis (Fig. S23). Additionally, the maintained atomic dispersion of Cu species in MOF-808-NaCu-275 (Fig. S24), but the formation of Cu NPs in MOF-808-Cu-275 (Fig. S25), has been proven by using TEM analysis. These results suggest that the presence of neighboring Na^+^ ions can effectively stabilize single-atom Cu sites within MOF-808, thereby preventing their aggregation under the hydrogenation conditions. This stabilization effect might be responsible for the outstanding methanol production performance at elevated temperatures.

**Figure 5.**
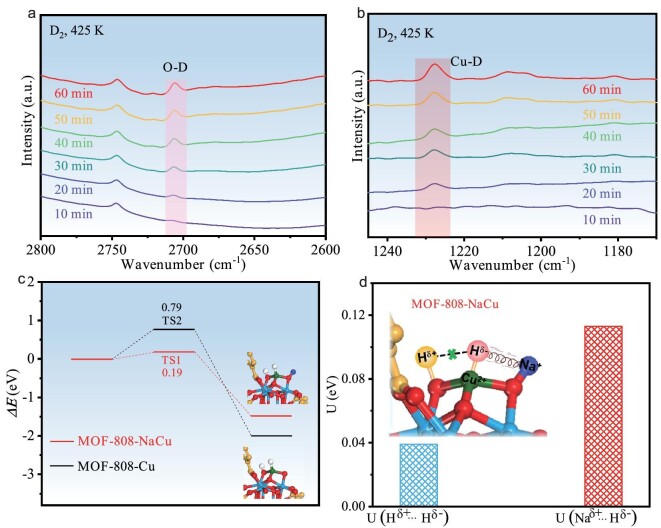
The generation and stabilization of H^δ−^ species over MOF-808-NaCu. (a) O–D signals and (b) Cu–D signals in *in situ* D_2_–DRIFT spectra for MOF-808-NaCu at 425 K. (c) The energy profiles of H_2_ dissociation in the heterolytic pathway on MOF-808-Cu and MOF-808-NaCu. (d) The electrostatic potential energy between the H^δ−^ and adjacent Na^+^ within MOF-808-NaCu (red) and the H^δ−^ and adjacent H^δ+^ within MOF-808-Cu (blue).

Then, the inherent mechanism for the stabilization effect induced by Na^+^-decorated microenvironment has been further investigated. Initially, active hydrogen species responsible for the reduction of Cu species under hydrogenation conditions are identified through *in situ* deuterium (D_2_)–DRIFT spectroscopy. Upon the introduction of D_2_ gas over MOF-808-NaCu, two new bands emerge at 2707 and 1227 cm^−1^, corresponding to surface vibrations of O–D and Cu–D, respectively (Fig. 5a and b) [43]. This observation strongly suggests the existence of a heterolytic D_2_ dissociation mechanism within MOF-808-NaCu, leading to the localization of D^δ−^ on Cu and D^δ+^ on adjacent O. Furthermore, this heterolytic dissociation pathway is supported by density functional theory (DFT) calculations, which indicate the heterolytic dissociation of H_2_ molecules facilitated by Cu–O bonds (Fig. 5c and d). According to previous reports [44], the reduction of Cu species typically follows the reductive elimination process from H^δ−^–Cu(II)–O to Cu(0)–OH^δ+^. In comparison with the low electrostatic interaction energy (U_E_) of 0.039 eV between H^δ+^ and H^δ−^ in MOF-808-Cu, MOF-808-NaCu displays significantly higher U_E_ between Na^+^ and H^δ−^ (0.113 eV) (Fig. 5d) on account of the higher Bader charge values (+0.878 a.u. for Na^+^ in MOF-808-NaCu vs. +0.618 a.u. for H^δ+^ in MOF-808-Cu). Such strong electrostatic attraction of the H^δ−^ on Cu(II) by adjacent Na^+^ significantly weakens the coulombic interaction between Cu(II) and H^δ−^ (similarly to a model of oscillating H^δ−^ between Cu(II) and Na^+^ cations), which would play a crucial role in suppressing the reductive elimination process, ultimately leading to the stabilization of Cu(II) sites (Fig. S26). On the other hand, the stabilization effect created by Na^+^ can also be contributed by the energetically favorable Na^+^···H^δ−^ attractive interaction; by contrast, if the reductive elimination pathway from H^δ−^–Cu(II)–O to Cu(0)–OH^δ+^ takes place, the energetically unfavorable Na^+^···H^δ+^ repulsive interaction would be generated. In summary, the unique stabilization effect induced by neighboring Na^+^ can be well explained by the strong interaction with H^δ−^ species, which impedes the reductive elimination of H^δ−^–Cu(II)–O and the formation of Cu(0)–OH^δ+^, thus resulting in enhanced stability of single-atom Cu sites in MOF-808-NaCu.

**Figure 6.**
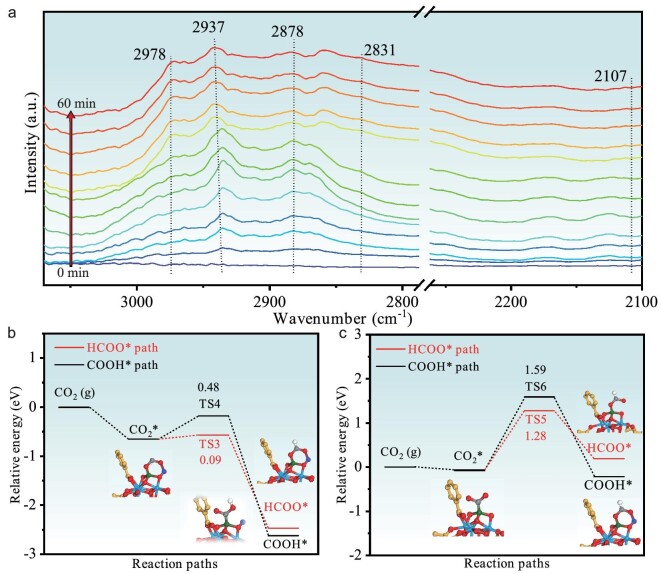
The investigation of the hydrogenation mechanism. (a) *In situ* DRIFTS spectra over MOF-808-NaCu under CO_2_/H_2_ mixtures (test conditions: CO_2_/H_2_ volume ratio of 1/3 with a flow rate of 16 mL min^−1^ at 225°C). Energy profiles of CO_2_ hydrogenation paths on (b) MOF-808-NaCu and (c) MOF-808-Cu, respectively (inset, the structures of intermediate geometries; blue, Zr; red, O; white, H; gray, C of CO_2_; purple, Na).

In order to obtain a more comprehensive understanding of the reaction mechanism, the surface species involved during the hydrogenation process have been monitored by adopting *in situ* DRIFTS under typical CO_2_/H_2_ mixtures at 498 K. When the gas mixture is introduced over MOF-808-NaCu-225, two new peaks show up at ∼2978 and ∼2878 cm^−1^, attributed to the characteristic features of adsorbed formate (HCOO*), and the infrared spectroscopy bands of the methoxy groups (H_3_CO*) can be simultaneously found at ∼2937 and ∼2831 cm^−1^ (Fig. 6a) [45]. Remarkably, the vibration peak at ∼2107 cm^−1^, typically associated with CO*, is not observed during the reaction. These findings unambiguously demonstrate that the dominant hydrogenation pathway over MOF-808-NaCu involves formate/methoxy intermediates, namely HCOO* and H_3_CO*. In contrast, the CO* peak (at ∼2107 cm^−1^) is readily detectable for MOF-808-Cu-225 involving Cu NPs generated *in situ* upon the introduction of reaction mixtures, suggesting the involvement of the COOH* (namely RWGS + CO-hydro) route (Fig. S27) [46,47]. To further confirm the distinct reaction pathways, *in situ* CO–DRIFTS spectra over MOF-808-NaCu-225 and MOF-808-Cu-225 are collected. Although significant CO signals can be detected over both catalysts upon introducing CO, this peak almost vanishes completely over MOF-808-NaCu-225 but remains unaltered over MOF-808-Cu-225 after argon purging for 30 min (Figs S28 and S29). These findings indicate a weak interaction between CO and single-atom Cu sites in MOF-808-NaCu, further supporting the absence of the RWGS + CO-hydro pathway on MOF-808-NaCu.

In addition, DFT calculations have been executed to verify the mechanism for CO_2_ hydrogenation proposed above. Remarkably, the formate-mediated pathway involving the HCOO* intermediate is found to be the preferred option with a lower energy barrier for both MOF-808-Cu and MOF-808-NaCu bearing single-atom Cu sites. This outcome offers a rational explanation for the predominant production of methanol at relatively low temperatures (<200°C), where single-atom Cu sites serve as the primary catalytic centers in both catalysts. Moreover, MOF-808-NaCu displays a very low energy barrier (0.09 eV, Fig. 6b) in the formate-mediated pathway when compared with the barrier over MOF-808-Cu (1.28 eV, Fig. 6c). This indicates the promotion effect on CO_2_ hydrogenation by Na^+^ microenvironment and also explains the higher methanol production activity over MOF-808-NaCu than MOF-808-Cu at relatively low temperatures (Fig. 4a and b). As the temperature rises, Cu NPs are generated *in situ* in MOF-808-Cu. Accordingly, the energy barrier for the formation of HCOO* (3.29 eV) is slightly higher than that for COOH* (2.96 eV) based on the Cu NPs (Fig. S30), implying the coexistence of both pathways, which is consistent with *in situ* DRIFTS results (Fig. 6a and Fig. S27). Overall, these results obtained through *in situ* DRIFT analysis and DFT calculation jointly indicate that the HCOO* pathway is the preferred route over Cu SACs (specifically, MOF-808-NaCu at all evaluated temperatures and MOF-808-Cu at temperatures of <200°C) for CO_2_ hydrogenation, while both HCOO* and COOH* routes coexist with catalysts involving Cu NPs (MOF-808-Cu at elevated temperatures).

## CONCLUSIONS

In summary, a facile synthetic strategy has been developed for the fabrication of single-atom Cu sites accompanied by adjacent Na^+^ ions on the Zr–oxo clusters of MOF-808. The resulting MOF-808-NaCu demonstrates exceptional performance in CO_2_ hydrogenation, achieving high methanol selectivity of >93% across the evaluated temperature range (from 150°C to 275°C) with a maximum methanol STY reaching 306 g⋅kg_cat_^−1^⋅h^−1^ at 275°C as well as long-term stability, far superior to those of the MOF-808-Cu counterpart (methanol STY of 28 g⋅kg_cat_^−1^⋅h^−1^ and selectivity of 35% at 275°C). Both the experimental and DFT calculation results suggest that the Na^+^-decorated microenvironment around Cu sites plays crucial roles, generating electrostatic interaction between the Na^+^ and H^δ−^ species to stabilize single-atom Cu sites and giving rise to significantly reduced energy barriers, which account for the improved selective CO_2_ hydrogenation. This work not only develops efficient and stable Cu SACs for selective CO_2_ hydrogenation, but also unveils the particular roles of an alkali-metal ion microenvironment in the stabilization of single-atom metal sites.

## Supplementary Material

nwae114_Supplemental_File

## References

[1] Zhong J, Yang X, Wu Z et al. State of the art and perspectives in heterogeneous catalysis of CO_2_ hydrogenation to methanol. ACS Catal 2020; 49: 14694–706. 10.1039/C9CS00614A 32067007

[2] Jiang X, Nie X, Guo X et al. Recent advances in carbon dioxide hydrogenation to methanol via heterogeneous catalysis. Chem Rev 2020; 120: 7984–8034. 10.1021/acs.chemrev.9b00723 32049507

[3] Hu J, Yu L, Deng J et al. Sulfur vacancy-rich MoS_2_ as a catalyst for the hydrogenation of CO_2_ to methanol. Nat Catal 2021; 4: 242–50. 10.1038/s41929-021-00584-3

[4] Wang K, Oe H, Nakaji Y et al. Carbon-neutral butadiene rubber from CO_2_. Chem 2021; 10: 419–26. 10.1016/j.chempr.2024.01.004

[5] He Z, Cui M, Qian Q et al. Synthesis of liquid fuel via direct hydrogenation of CO_2_. Proc Natl Acad Sci USA 2019; 116: 12654–9. 10.1073/pnas.1821231116 31182598 PMC6600929

[6] Kattel S, Ramírez PJ, Chen JG et al. Active sites for CO_2_ hydrogenation to methanol on Cu/ZnO catalysts. Science 2017; 355: 1296–9. 10.1126/science.aal3573 28336665

[7] Wang J, Li G, Li Z et al. A highly selective and stable ZnO-ZrO_2_ solid solution catalyst for CO_2_ hydrogenation to methanol. Sci Adv 2017; 3: e1701290. 10.1126/sciadv.1701290 28989964 PMC5630239

[8] Beck A, Zabilskiy M, Newton MA et al. Following the structure of copper-zinc-alumina across the pressure gap in carbon dioxide hydrogenation. Nat Catal 2021; 4: 488–97. 10.1038/s41929-021-00625-x

[9] Kordus D, Jelic J, Luna ML et al. Shape-dependent CO_2_ hydrogenation to methanol over Cu_2_O nanocubes supported on ZnO. J Am Chem Soc 2023; 145: 3016–30. 10.1021/jacs.2c11540 36716273 PMC9912329

[10] Zhou H, Chen Z, López AV et al. Engineering the Cu/Mo_2_CT_x_ (MXene) interface to drive CO_2_ hydrogenation to methanol. Nat Catal 2021; 4: 860–71. 10.1038/s41929-021-00684-0

[11] Zhao H, Yu R, Ma S et al. The role of Cu_1_-O_3_ species in single-atom Cu/ZrO_2_ catalyst for CO_2_ hydrogenation. Nat Catal 2022; 5: 818–31. 10.1038/s41929-022-00840-0

[12] Yang T, Mao X, Zhang Y et al. Coordination tailoring of Cu single sites on C_3_N_4_ realizes selective CO_2_ hydrogenation at low temperature. Nat Commun 2021; 12: 6022. 10.1038/s41467-021-26316-6 34654822 PMC8519910

[13] Kaiser SK, Chen Z, Akl DF et al Single-atom catalysts across the periodic table. Chem Rev 2020; 120: 11703–809. 10.1021/acs.chemrev.0c00576 33085890

[14] Liu L, Corma A. Metal catalysts for heterogeneous catalysis: from single atoms to nanoclusters and nanoparticles. Chem Rev 2018; 118: 4981–5079. 10.1021/acs.chemrev.7b00776 29658707 PMC6061779

[15] Ji S, Chen Y, Wang X et al. Chemical synthesis of single atomic site catalysts. Chem Rev 2020; 120: 11900–55. 10.1021/acs.chemrev.9b00818 32242408

[16] Qin R, Zhou L, Liu P et al. Alkali ions secure hydrides for catalytic hydrogenation. Nat Catal 2020; 3: 703–9. 10.1038/s41929-020-0481-6

[17] Qiao B, Wang A, Yang X et al. Single-atom catalysis of CO oxidation using Pt_1_/FeO_x_. Nat Chem 2011; 3: 634–41. 10.1038/nchem.1095 21778984

[18] Shan J, Ye C, Zhu C et al. Integrating interactive noble metal single-atom catalysts into transition metal oxide lattices. J Am Chem Soc 2022; 144: 23214–22. 10.1021/jacs.2c11374 36475661

[19] Deng X, Qin B, Liu R et al. Zeolite-encaged isolated platinum ions enable heterolytic dihydrogen activation and selective hydrogenations. J Am Chem Soc 2021; 143: 20898–906. 10.1021/jacs.1c09535 34855383

[20] Li Q, Yan G, Vlachos DG. Theoretical insights into H_2_ activation over anatase TiO_2_ supported metal adatoms. ACS Catal 2024; 14: 886–96.

[21] Li X, Pereira-Hernández XI, Chen Y et al. Functional CeO_*x*_ nanoglues for robust atomically dispersed catalysts. Nature 2022; 611: 284–8. 10.1038/s41586-022-05251-6 36289341

[22] Sui J, Liu H, Hu S et al. A general strategy to immobilize single-atom catalysts in metal–organic frameworks for enhanced photocatalysis. Adv Mater 2022; 34: 2109203. 10.1002/adma.202109203 34883530

[23] Li Z, Li B, Li Q. Single-atom Nano-Islands (SANIs): a robust atomic–nano system for versatile heterogeneous catalysis applications. Adv Mater 2023; 35: 2211103. 10.1002/adma.202211103 36967534

[24] Furukawa H, Cordova KE, O'Keeffe M et al. The chemistry and applications of metal-organic frameworks. Science 2013; 341: 1230444. 10.1126/science.1230444 23990564

[25] Ding M, Flaig RW, Jiang H-L et al. Carbon capture and conversion using metal–organic frameworks and MOF-based materials. Chem Soc Rev 2019; 48: 2783–828. 10.1039/C8CS00829A 31032507

[26] Stanley PM, Haimerl J, Shustova N B et al Merging molecular catalysts and metal–organic frameworks for photocatalytic fuel production. Nat Chem 2022; 14: 1342–56. 10.1038/s41557-022-01093-x 36443532

[27] Li B, Wen H-M, Cui Y et al. Emerging multifunctional metal-organic framework materials. Adv Mater 2016; 28: 8819–60. 10.1002/adma.201601133 27454668

[28] Zhao X, Wang Y, Li DS et al. Metal-organic frameworks for separation. Adv Mater 2018; 30: 1705189. 10.1002/adma.201705189 29582482

[29] Navalon S, Dhakshinamoorthy A, Alvaro M et al. Metal-organic frameworks as photocatalysts for solar-driven overall water splitting. Chem Rev 2023; 123: 445–90. 10.1021/acs.chemrev.2c00460 36503233 PMC9837824

[30] Guo J, Qin Y, Zhu Y et al. Metal–organic frameworks as catalytic selectivity regulators for organic transformations. Chem Soc Rev 2021; 50: 5366–96. 10.1039/D0CS01538E 33870965

[31] Manna K, Ji P, Greene FX et al. Metal–organic framework nodes support single-site magnesium–alkyl catalysts for hydroboration and hydroamination reactions. J Am Chem Soc 2016; 138: 7488–91. 10.1021/jacs.6b03689 27282364

[32] Fang G, Wei F, Lin J et al. Retrofitting Zr-Oxo nodes of UiO-66 by Ru single atoms to boost methane hydroxylation with nearly total selectivity. J Am Chem Soc 2023; 145: 13169–80. 10.1021/jacs.3c02121 37279334

[33] Jiao L, Jiang H-L. Metal-organic framework-based single-atom catalysts for energy applications. Chem 2019; 5: 786–804. 10.1016/j.chempr.2018.12.011

[34] Rungtaweevoranit B, Baek J, Araujo J R et al Copper nanocrystals encapsulated in Zr-based metal–organic frameworks for highly selective CO_2_ hydrogenation to methanol. Nano Lett 2016; 16: 7645–9. 10.1021/acs.nanolett.6b03637 27960445

[35] Gutterod ES, Lazzarini A, Fjermestad T et al. Hydrogenation of CO_2_ to methanol by Pt nanoparticles encapsulated in UiO-67: deciphering the role of the metal–organic framework. J Am Chem Soc 2020; 142: 999–1009. 10.1021/jacs.9b10873 31794194

[36] An B, Zhang J, Cheng K et al. Confinement of ultrasmall Cu/ZnO_x_ nanoparticles in metal–organic frameworks for selective methanol synthesis from catalytic hydrogenation of CO2. J Am Chem Soc 2017; 139: 3834–40. 10.1021/jacs.7b00058 28209054

[37] Kobayashi H, Taylor JM, Mitsuka Y et al. Charge transfer dependence on CO_2_ hydrogenation activity to methanol in Cu nanoparticles covered with metal–organic framework systems. Chem Sci 2019; 10: 3289–94. 10.1039/C8SC05441J 30996914 PMC6429599

[38] Zhu Y, Zheng J, Ye J et al. Copper-zirconia interfaces in UiO-66 enable selective catalytic hydrogenation of CO_2_ to methanol. Nat Commun 2020; 11: 5849. 10.1038/s41467-020-19438-w 33208734 PMC7674450

[39] Hou S-L, Dong J, Zhao X-Y et al. Thermocatalytic conversion of CO_2_ to valuable products activated by noble-metal-free metal-organic frameworks. Angew Chem Int Ed 2023; 62: e202305213. 10.1002/anie.202305213 37170958

[40] Ling L-L, Yang W, Yan P et al. Light-assisted CO_2_ hydrogenation over Pd_3_Cu@UiO-66 promoted by active sites in close proximity. Angew Chem Int Ed 2022; 61: e202116396. 10.1002/anie.202116396 34931422

[41] An B, Li Z, Song Y et al. Cooperative copper centres in a metal–organic framework for selective conversion of CO_2_ to ethanol. Nat Catal 2019; 2: 709–17. 10.1038/s41929-019-0308-5

[42] Yang M, Liu J, Lee S et al. A common single-site Pt(II)-O(OH)_x_-species stabilized by sodium on ‘active’ and ‘inert’ supports catalyzes the water-gas shift reaction. J Am Chem Soc 2015; 137: 3470–3. 10.1021/ja513292k 25746682

[43] Deng Q, Li X, Gao R et al. Hydrogen-catalyzed acid transformation for the hydration of alkenes and epoxy alkanes over Co-N frustrated lewis pair surfaces. J Am Chem Soc 2021; 143: 21294–301. 10.1021/jacs.1c08259 34874721

[44] Shun K, Mori K, Masuda S et al. Revealing hydrogen spillover pathways in reducible metal oxides. Chem Sci 2022; 13: 8137–47. 10.1039/D2SC00871H 35919430 PMC9278487

[45] Feng Z, Tang C, Zhang P et al. Asymmetric sites on the ZnZrO_*x*_ catalyst for promoting formate formation and transformation in CO_2_ hydrogenation. J Am Chem Soc 2023; 145: 12663–72. 10.1021/jacs.3c02248 37261391

[46] Yang C, Pei C, Luo R et al. Strong electronic pxide–support interaction over In_2_O_3_/ZrO_2_ for highly selective CO_2_ hydrogenation to methanol. J Am Chem Soc 2020; 142: 19523–31. 10.1021/jacs.0c07195 33156989

[47] Yu J, Yang M, Zhang J et al. Stabilizing Cu^+^ in Cu/SiO_2_ catalysts with a shattuckite-like structure boosts CO_2_ hydrogenation into methanol. ACS Catal 2020; 10: 14694–706. 10.1021/acscatal.0c04371

