# Supplementary material for: Promoted hydrogenation of CO2 to methanol over single-atom Cu sites with Na+-decorated microenvironment
Source: Natl Sci Rev. 2024 Mar 22;11(6):nwae114. doi: 10.1093/nsr/nwae114 (PMC11073544; doi:10.1093/nsr/nwae114)
Supplement: nwae114_Supplemental_File [file nwae114_supplemental_file.pdf]

Supporting Information

Promoted hydrogenation of CO_2 to methanol over single-atom Cu sites with Na^+ decorated microenvironment

Li-Li Ling[1,†], Xinyu Guan[1,†], Xiaoshuo Liu[2,3], Xiao-Mei Lei[1], Zhongyuan Lin[1] and Hai-Long Jiang[1,*]

[1]Hefei National Research Center for Physical Sciences at the Microscale, Department of Chemistry, University of Science and Technology of China, Hefei, Anhui 230026, P. R. China.

[2]School of Energy and Power Engineering, North China Electric Power University, Baoding, Hebei 071003, P. R. China.

[3]School of Energy and Environment, Southeast University, Nanjing, Jiangsu 210096, P. R. China.

Corresponding author. Email: jianglab@ustc.edu.cn (H.-L.J).

[†]These authors contributed equally to this work.

1. Materials and Characterizations

All chemical materials and solvents were obtained from commercial sources and used directly without any further purification otherwise stated: $ZrOCl_2 \cdot 8H_2O$ (Aladdin Industrial Inc., 98%), trimesic acid (H_3BTC, Aladdin Industrial Inc., 98%), sodium tert-butoxide (Energy Chemical, 1.0 mol L^{-1} in THF), tetrakis(acetonitrile)copper tetrafluoroborate (Energy Chemical, 97%), N, N-dimethylformamide (Sinopharm Chemical Reagent Co., Ltd., AR), formic acid (Sinopharm Chemical Reagent Co., Ltd., AR), tetrahydrofuran (Energy Chemical., 99.9%) and quartz sand (Sinopharm Chemical Reagent Co., Ltd., AR).

Powder X-ray diffraction (PXRD) measurements were conducted on a Japan Rigaku Miniflex 600 rotation anode X-ray diffractometer equipped with Cu Kα radiation (λ = 1.5418 Å) over the range of 2θ = 2.0-50.0 ° with a step size of 0.03 ° and 3 s per step. The contents of Na and Cu were quantified by an Optima 7300 DV inductively coupled plasma orbital emission spectrometer (ICP-OES). The N_2 sorption isotherms at 77 K were taken on a Micromeritics ASAP 2020 instrument. X-ray photoelectron spectroscopy (XPS) data were collected on an ESCALAB 250 high-performance electron spectrometer using monochromatized Al Kα (hν = 1486.7 eV) as the excitation source. Scanning electron microscopy (SEM) images were obtained on a Carl Zeiss Supra 40 scanning electron microscope. Transmission electron microscopy (TEM) images were obtained on a JEM-2010F instrument. Temperature-programmed desorption of CO_2 (CO_2-TPD) was performed on a Micromeritics AutoChem II 2920 instrument. Diffuse reflectance infrared Fourier transform (DRIFT) measurements were performed on a Nicolet™ iS™ 10 FTIR spectrometer with an MCT detector. The catalytic reaction products were identified by gas chromatography (GC, Agilent 8860).

The XANES and EXAFS analyses for the Cu K-edge were performed at the 1W1B station of BSRF and 14WB1 beamline of SSRF. All data of catalysts and metal foils were obtained via transmission mode. Data analysis was implemented on Athena and Artemiss software packages. Energy calibration was performed based on metal foils.

For EXAFS modeling, EXAFS of the metal foil was fitted, and the obtained amplitude reduction factor S02 value was set for the EXAFS fitting to determine.

2. Materials Preparation

Synthesis of MOF-808: Generally, MOF-808 was prepared using the literature method.[1] Typically, 194 mg $ZrOCl_2 \cdot 8H_2O$ and 42 mg H_3BTC were ultrasonically dispersed in a mixed solution of 6 mL HCOOH and 6 mL DMF. The mixed solution was transferred into a 20 mL vessel and allowed to react at 100 ℃ for 24 h. After cooling down to room temperature, the white solid was centrifuged and washed with DMF and acetone for three times respectively. The as-synthesized MOF-808 was thoroughly immersed and exchanged by acetone and activated at 60 ℃ for 12 h under vacuum. The dry sample was then treated with 1 M HCl at 90 ℃ for 12 h and washed with water and acetone for several times. The activated MOF-808 samples were kept in a 60 ℃ vacuum oven for subsequent modification and characterization.

Synthesis of MOF-808-Cu: Typically, 50 mg MOF-808 was ultrasonically dispersed in 25 mL anhydrous THF, followed by adding 12.5 mg $Cu(CH_3CN)_4BF_4$ under ultrasonication. The reaction mixture was then stirred at room temperature for 24 h. The blue solid was collected by centrifugation, washed with DMF and acetone for several times and dried in a 60 ℃ vacuum oven to afford MOF-808-Cu.

Synthesis of MOF-808-Na: Typically, 100 mg MOF-808 was ultrasonically dispersed in 50 mL anhydrous THF at 0 ℃, followed by adding 400 μL of sodium tert-butoxide (1 $mol \cdot L^{-1}$ in THF solution). The reaction mixture was then stirred for 40 minutes under 0 ℃. The white solid was collected by centrifugation and washed with anhydrous THF for six times. The as-prepared samples were then dried in a 60 ℃ vacuum oven to afford MOF-808-Na.

Synthesis of MOF-808-NaCu: Typically, 50 mg MOF-808-Na was ultrasonically dispersed in 25 mL anhydrous THF, followed by adding 12.5 mg of $Cu(CH_3CN)_4BF_4$ under ultrasonication. The reaction mixture was then stirred at room temperature for 24 h. The solid sample was collected by centrifugation, washed with anhydrous THF

for six times and dried in a 60 ℃ vacuum oven to afford MOF-808-NaCu.

Synthesis of MOF-808-KCu: MOF-808-KCu was obtained in a similar procedure to MOF-808-NaCu, except that potassium tert-butoxide (1 $mol \cdot L^{-1}$ in THF solution) was used instead of sodium tert-butoxide.

Synthesis of MOF-808-CsCu: MOF-808-NaCu (50 mg) was added to a saturated solution of CsF (63 mg, 6 equiv. to Na) in 50 mL of THF. The mixture was then stirred for 24 h before the solid was collected by centrifugation, and washed six times with THF to obtain MOF-808-CsCu.

3. Catalytic Performance for CO_2 Hydrogenation.

The CO_2 hydrogenation reaction was carried out in a tubular fixed-bed continuous-flow reactor equipped with GC. Before the reaction, the catalyst (50 mg, diluted with 0.2 g of quartz sand) was pretreated in a N_2 stream (1 bar and 30 $ml \cdot min^{-1}$) at a given temperature. The reaction was conducted under reaction conditions of 3.5 MPa, 150 to 275 ℃, $V(H_2)/V(CO_2)/V(Ar)$ = 72:24:4 and weight hourly space velocity (WHSV) = 60,000 $mL \cdot g^{-1} \cdot h^{-1}$. The exit gas from the reactor was maintained at 120 ℃ and immediately transported to the sample valve of the GC (Agilent 8860), which was equipped with thermal conductivity (TCD) and flame ionization detectors (FIDs). All data were collected within 3 hours after the reaction started (unless otherwise specified). The CO_2 conversion and CH_3OH selectivity were obtained from the GC data.

Conversion of CO_2, selectivity and space time yield (STY) of methanol denoted as $Conv_{CO2}$, Sel_{MeOH} and MeOH STY. The $Conv_{CO2}$, Sel_{MeOH} and MeOH STY were defined in the following equation:

$$Conv_{CO2} = 1 - \left(\frac{A_{CO_2,\ out}}{A_{CO_2,\ in}} \bullet \frac{A_{Ar,\ in}}{A_{Ar,\ out}}\right)$$

$A_{CO2,\ in}$ and $A_{Ar,\ in}$ represent the peak areas of the corresponding gases in the TCD chromatographs of the gas (CO_2/H_2) feed before catalysis, respectively.

$A_{CO2,\ out}$ and $A_{Ar,\ out}$ represent the peak areas of the exit gas composition during catalysis, respectively.

$$Sel_{MeOH} = \frac{n_{CH_3OH}}{n_{CO} + n_{CH_4} + n_{CH_3OH}}$$

$$\text{MeOH STY } (g_{CH3OH} \cdot kg_{cat}^{-1} \cdot h^{-1}) = \frac{FCO_2 * Conv_{CO2} * Sel_{MeOH} * 32 * 60}{22.4 * m_{cat}}$$

n_{CH3OH}, n_{CO} and n_{CH4} represent the molar quantities of the CH_3OH, CO and CH_4, respectively;

F_{CO2} represents the gas flow rate;

m_{cat} represents the weight of the catalyst.

A long-term catalytic test for CO_2 hydrogenation was conducted under similar reaction conditions but with the WHSV reduced to 12,000 $mL \cdot g^{-1} \cdot h^{-1}$.

4. DFT Computational Details

General procedure for DFT calculation. Spin-polarization DFT calculations were carried out in the Vienna Ab initio Simulation Package (VASP5.4.4), using the Perdew-Burke-Ernzerhof (PBE) functional and projector augmented wave (PAW) methods.[2-4] The DFT-D3 proposed by Grimme et al. was determined to correct van der Waals interactions.[5] The kinetic energy cutoff was adopted as 450 eV. Restricted geometry optimizations were carried out in gamma point k-mesh accuracy, with the convergence standard of 0.05 eV $Å^{-1}$. A climbing image nudged elastic band method developed by Henkelman et al. was utilized to find the transition state structures.[6] To accurately describe the catalytic reaction in accurate interaction sites, the simplified cluster was modeled in a relatively large cell, for which all 1,3,5-benzene tricarboxylate (BTC^{3-}) linkers were substituted by the formic acid structures, and corresponding atoms were constrained in DFT calculations to achieve the same boundary environment as periodic MOF-808 materials.[7] The Cu nanoparticle was approximately simulated using a three-layer 4 × 4 supercell of Cu (1 1 1) low index crystal plane with a 15 Å vacuum layer.[8] In the Cu slab model, bottom atoms were all frozen and only surface absorbed gaseous molecules and top two layers Cu atoms were optimized.

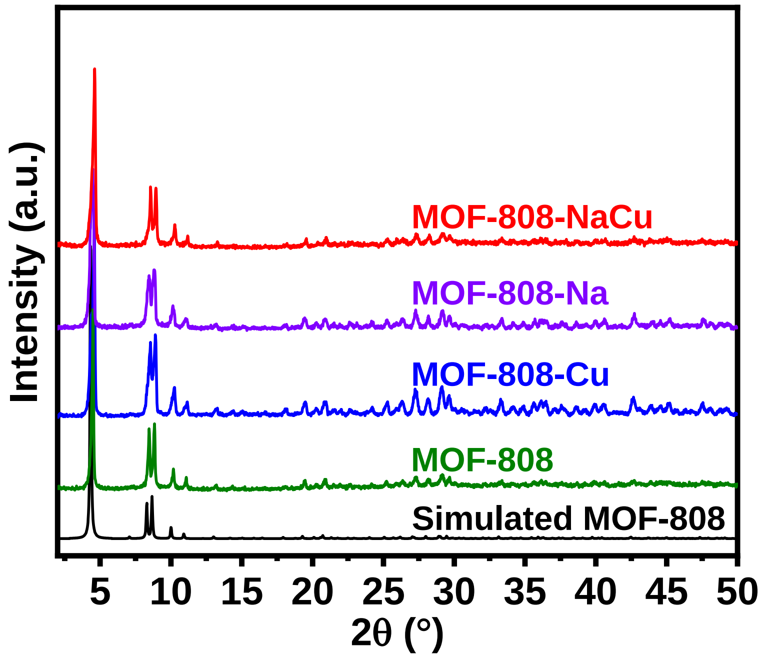

Figure S1. Powder XRD patterns of MOF-808-NaCu (red), MOF-808-Na (violet), MOF-808-Cu (blue) and MOF-808 (olive). All these modified MOF structures exhibit very similar patterns to that of the pristine MOF-808, indicating their well retained structures.

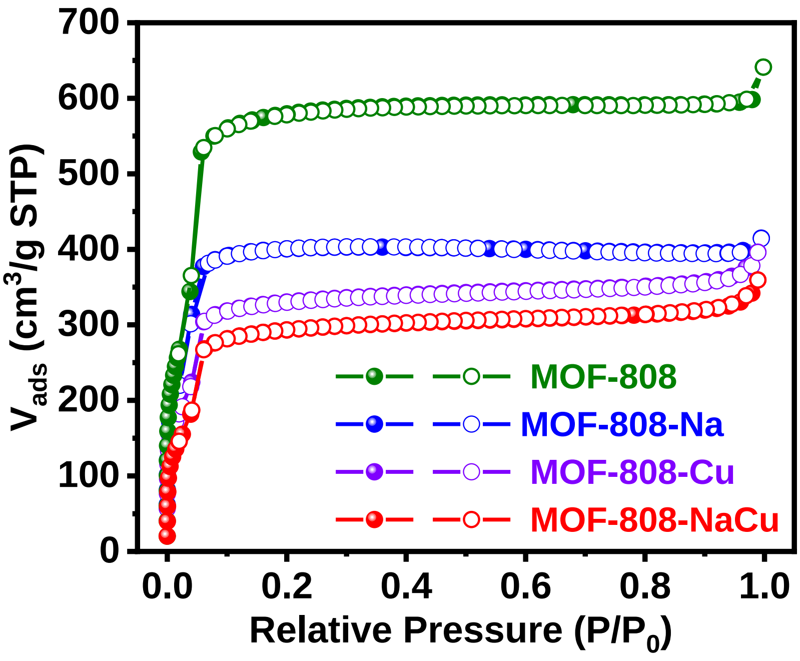

Figure S2. Nitrogen sorption isotherms at 77 K for MOF-808-NaCu (red), MOF-808-Cu (violet), MOF-808-Na (blue) and MOF-808 (olive).

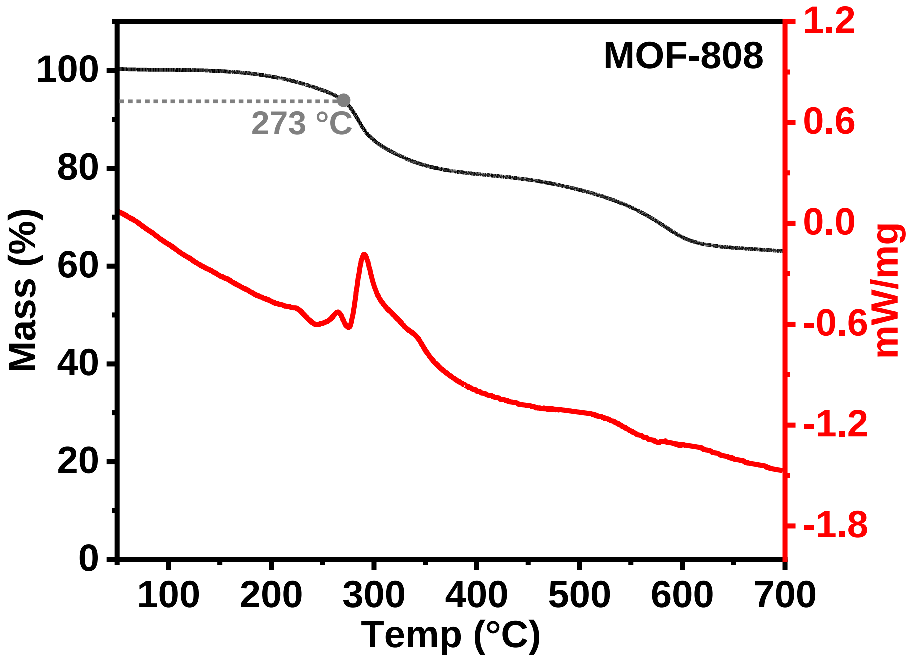

Figure S3. TGA curve of MOF-808 in the 50-700 °C range under N_2 atmosphere.

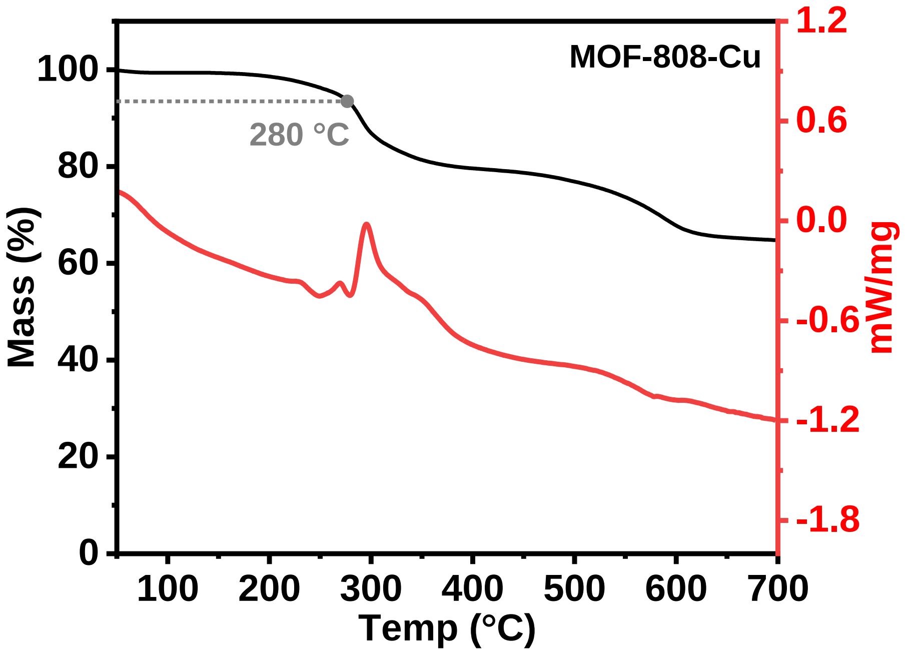

Figure S4. TGA curve of MOF-808-Cu in the 50-700 °C range under N_2 atmosphere.

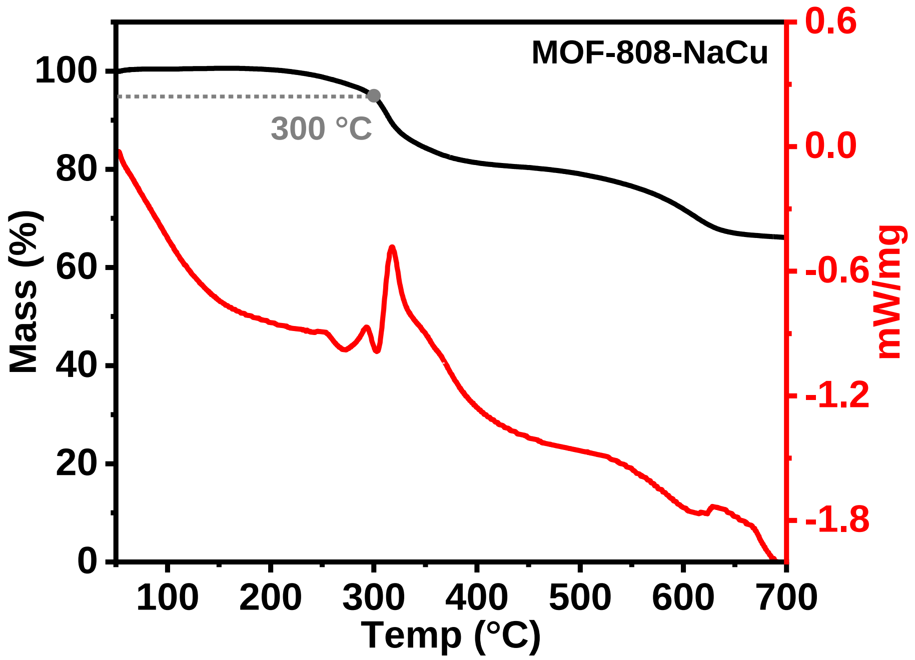

Figure S5. TGA curve of MOF-808-NaCu in the 50-700 °C range under N_2 atmosphere.

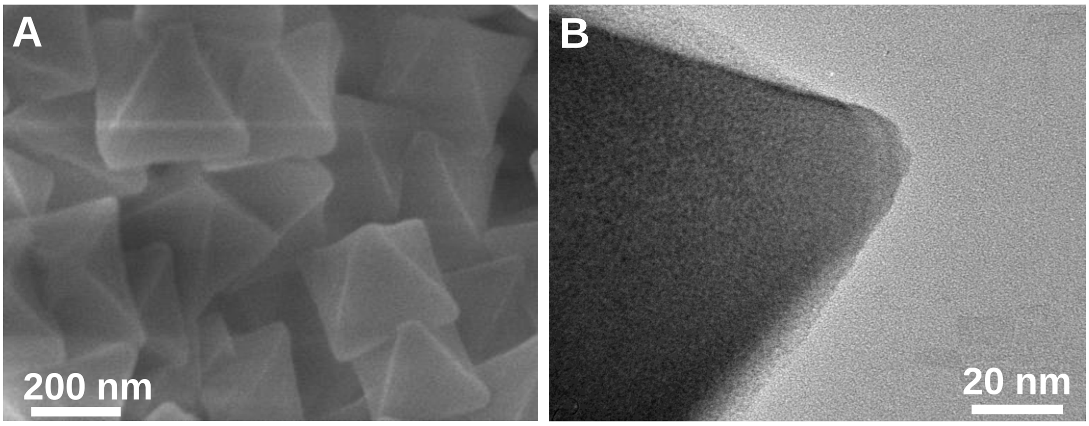

Figure S6. Microscopic characterizations. (*A*) SEM and (*B*) TEM images of MOF-808-Cu. No nanoparticles can be observed.

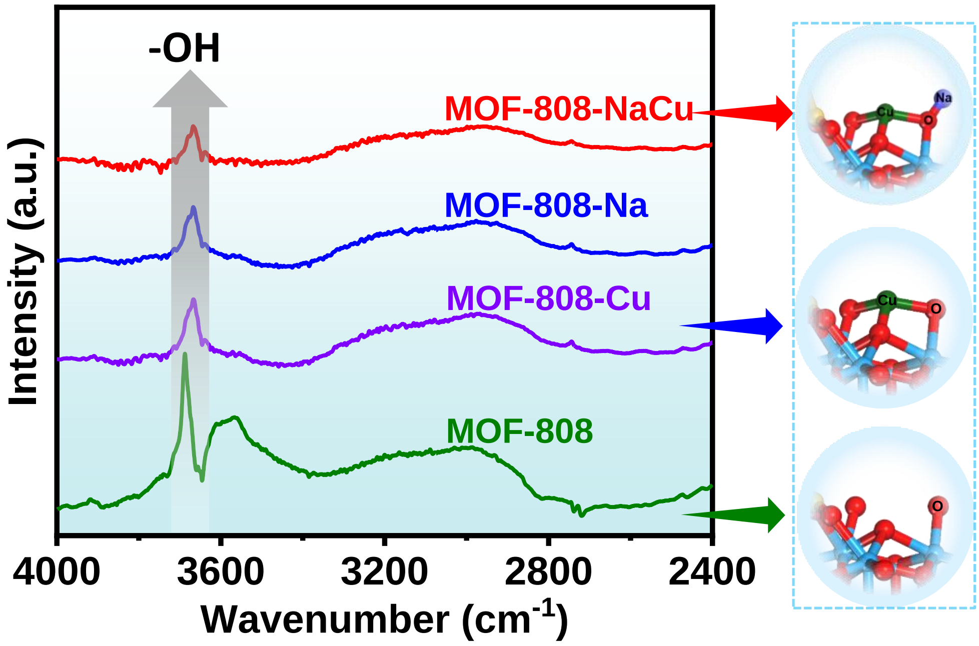

Figure S7. The DRIFTS spectra of MOF-808-NaCu (red), MOF-808-Na (blue), MOF-808-Cu (violet) and MOF-808 (olive). The intensity of adsorption peak associated with -OH/OH_2 groups (at approximately 3670 cm^{-1}) exhibits obvious decrease following the introduction of Na^+/Cu^{2+} loading, indicating the interaction with -OH/OH_2 groups (test conditions: N_2 with a flow rate of 16 mL·min^{-1} at 120 °C).

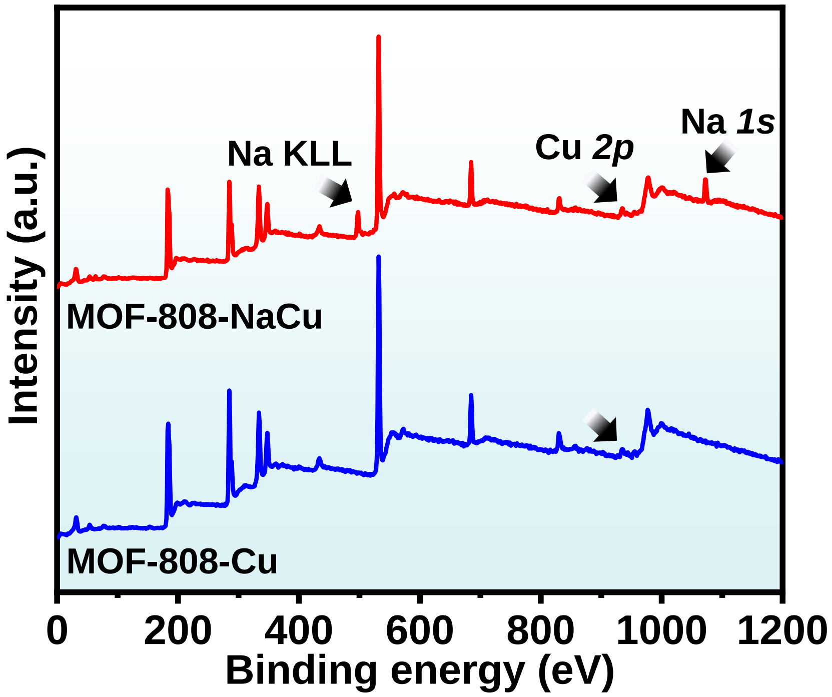

Figure S8. The XPS survey spectra of MOF-808-NaCu (red) and MOF-808-Cu (blue).

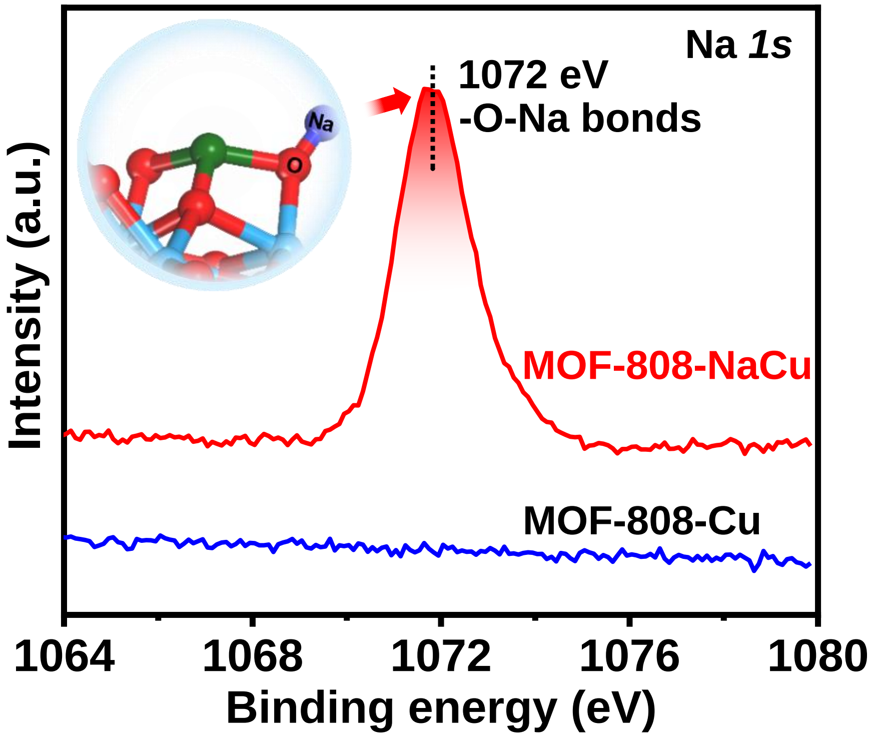

Figure S9. The Na *1s* XPS spectra of MOF-808-NaCu (red) and MOF-808-Cu (blue). The distinct Na *1s* peak appears at 1072 eV, indicating the presence of the O-Na bonding in MOF-808-NaCu.

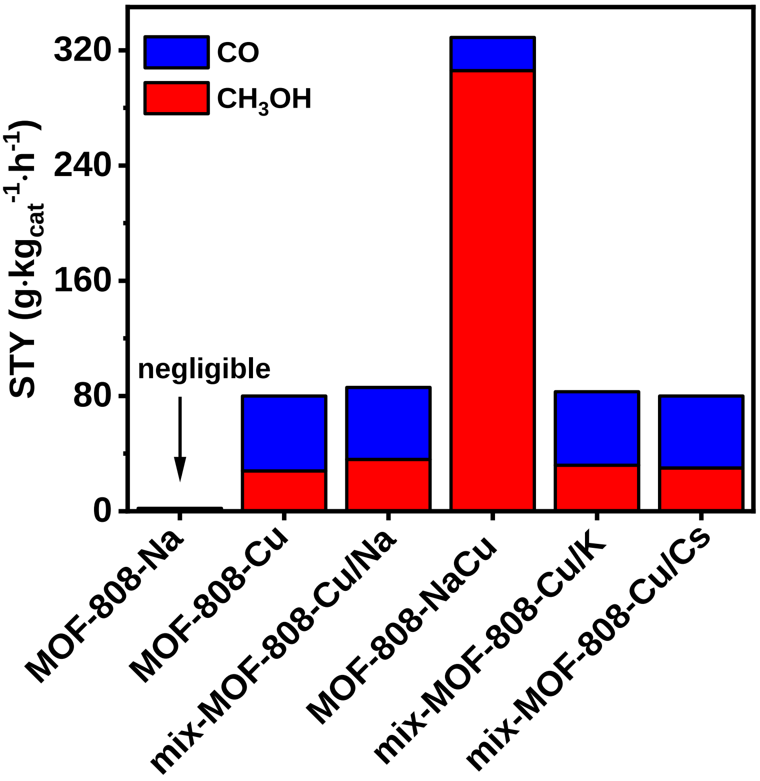

Figure S10. Catalytic performance of various catalysts in CO_2 hydrogenation (reaction conditions for the catalytic test: volume ratio of CO_2/H_2: 1:3, pressure: 3.5 MPa, temperature: 275 ℃).

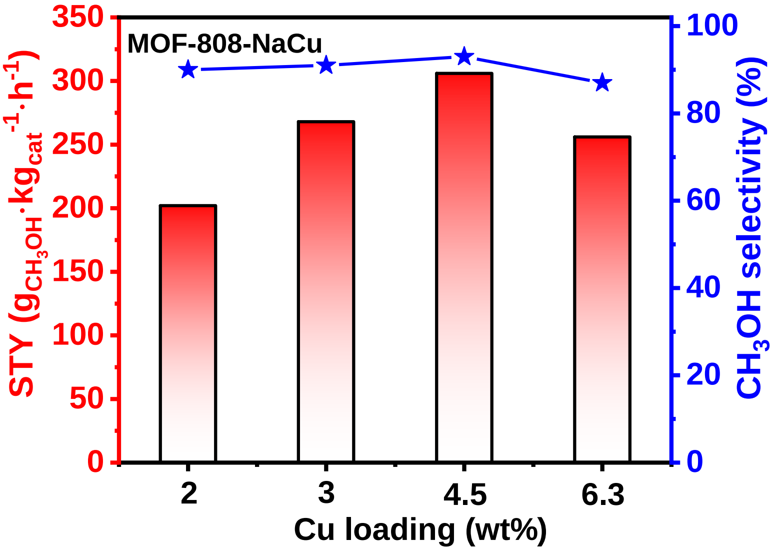

Figure S11. Catalytic performance of MOF-808-NaCu with varying levels of Cu loading (reaction conditions: volume ratio of CO_2/H_2: 1:3, pressure: 3.5 MPa, temperature: 275 ℃).

It was observed that both STY and selectivity of methanol improved as the Cu loading in MOF-808-NaCu increase from 2 wt% to 4.5 wt%. The slight decrease in STY and selectivity of methanol at a Cu loading of 6.3% may be attributed to the diminished accessibility of Cu sites due to the high atomic density.

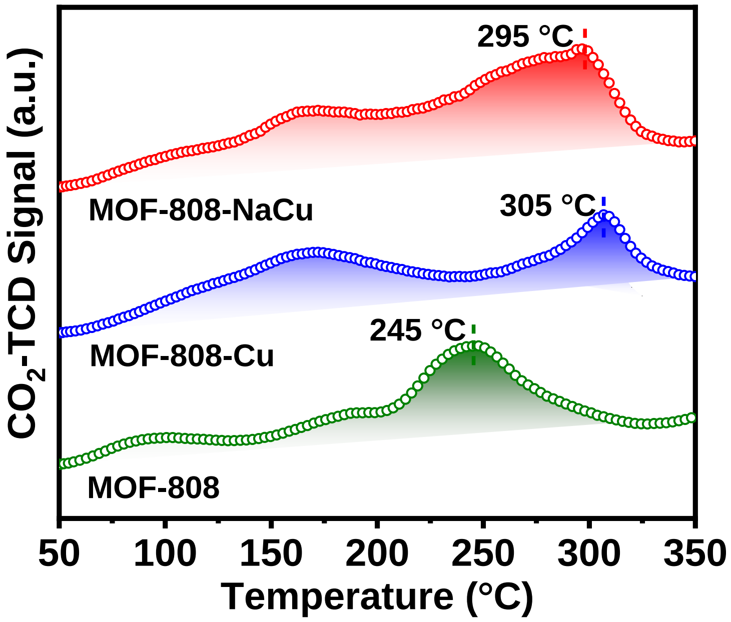

Figure S12. The CO_2-TPD curves of MOF-808-NaCu (red), MOF-808-Cu (blue) and MOF-808 (olive). Compared to the peak at 245 ℃ of MOF-808, the CO_2 desorption peaks of MOF-808-Cu and MOF-808-NaCu shift to higher temperatures of 305 ℃ and 295 ℃, respectively, suggesting that Cu is the potential CO_2 binding site.

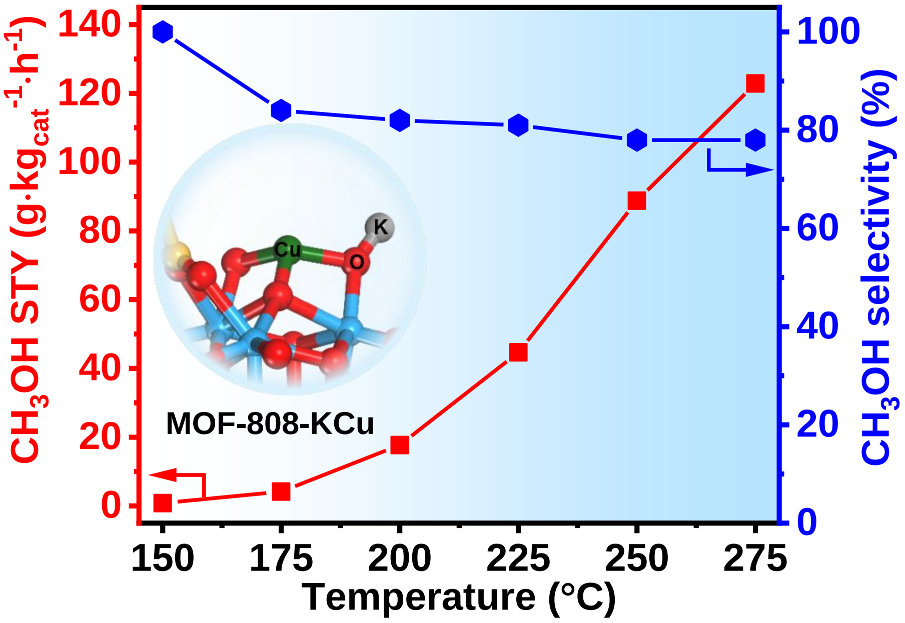

Figure S13. Catalytic performance of MOF-808-KCu in CO_2 hydrogenation. The methanol production rate (123 g kg_{cat}^{-1} h^{-1}) and selectivity (78%) over MOF-808-KCu are much enhanced compared with MOF-808-Cu at 275 ℃. Reaction conditions: CO_2/H_2 volume ratio of 1/3 with reaction pressure of 3.5 MPa and reaction temperatures from 150 to 275 ℃.

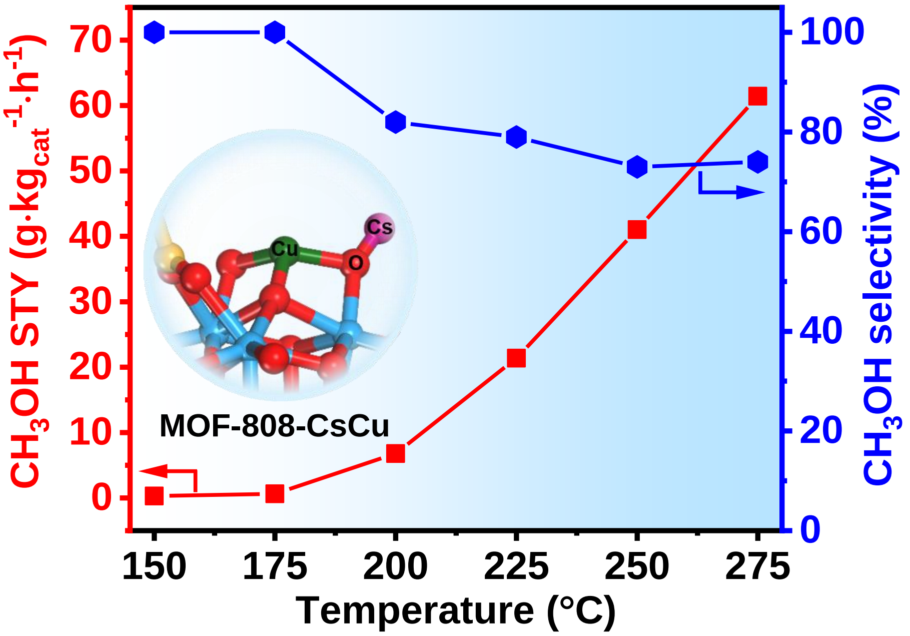

Figure S14. Catalytic performance of MOF-808-CsCu in CO_2 hydrogenation. The methanol production rate (64 g kg_{cat}^{-1} h^{-1}) and selectivity (74%) over MOF-808-CsCu are much enhanced compared with MOF-808-Cu at 275 ℃. Reaction conditions: CO_2/H_2 volume ratio of 1/3 with reaction pressure of 3.5 MPa and reaction temperature from 150 to 275 ℃.

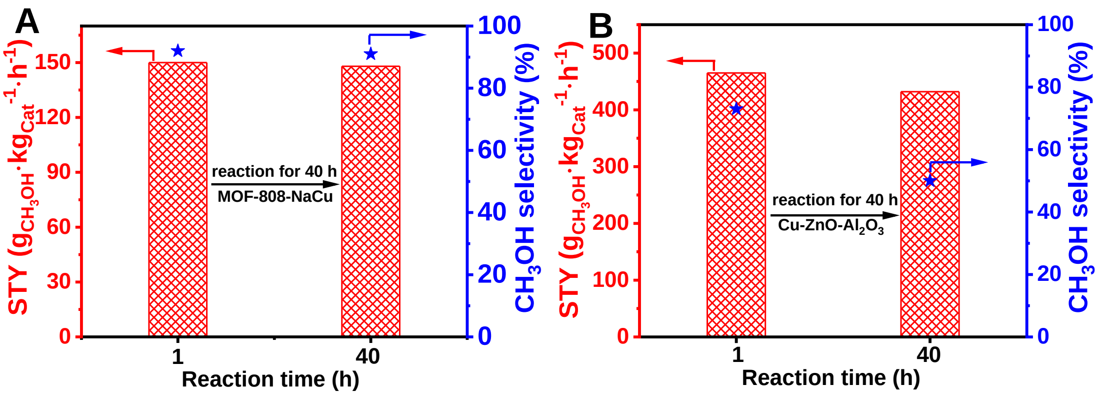

Figure S15. Catalytic performance (*A*) MOF-808-NaCu and (*B*) Cu-ZnO-Al_2O_3 in CO_2 hydrogenation. Reaction conditions: CO_2/H_2 volume ratio of 1/3 with reaction pressure of 3.5 MPa and reaction temperatures of 250 ℃.

While Cu-ZnO-Al_2O_3 catalyst exhibits a relatively high STY (465 g·kg_{cat}^{-1}·h^{-1}), it gives a methanol selectivity as low as 73%. More importantly, its stability is inadequate under the operating conditions, resulting in significant CO byproducts (432 g·kg_{cat}^{-1}·h^{-1}) and low methanol selectivity (50%) after 40 h, possibly due to the potential sintering of Cu species under the relative high temperatures.

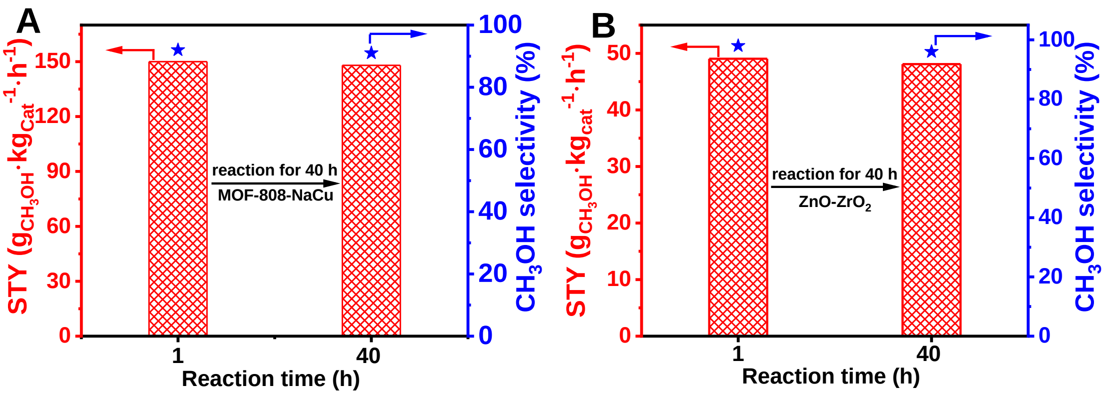

Figure S16. Catalytic performance (*A*) MOF-808-NaCu and (*B*) ZnO-ZrO_2 in CO_2 hydrogenation. Reaction conditions: CO_2/H_2 volume ratio of 1/3 with reaction pressure of 3.5 MPa and reaction temperatures of 250 ℃.

While the ZnO-ZrO_2 catalyst presents good methanol selectivity (96%) and stability, it exhibits considerably lower methanol activity (48.1 g·kg_{cat}^{-1}·h^{-1}), in comparison to MOF-808-NaCu.

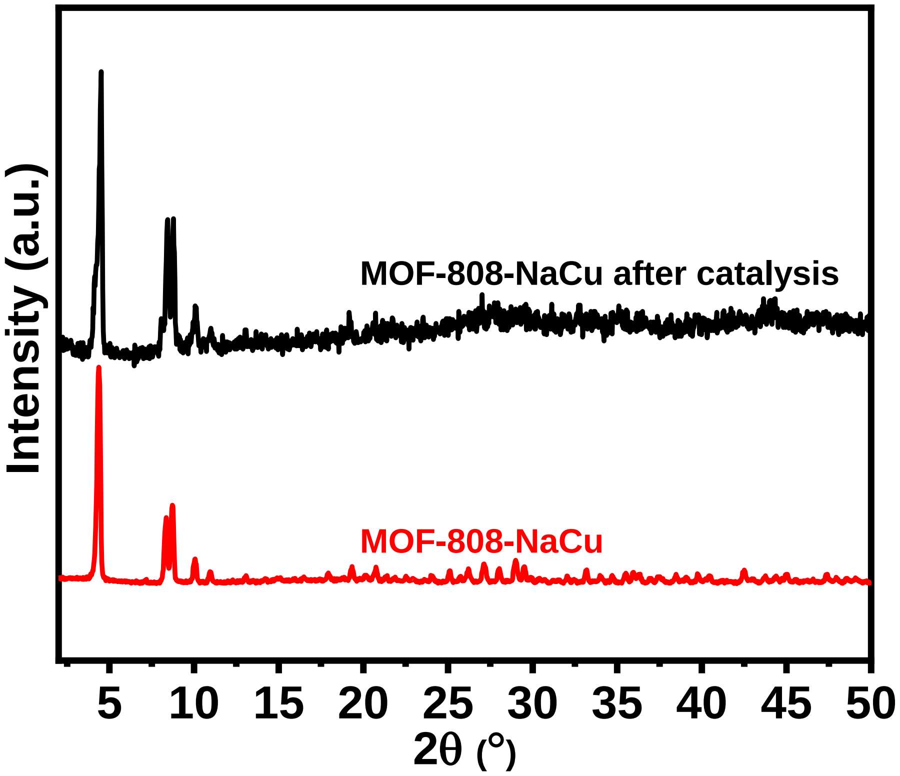

Figure S17. Powder XRD patterns of MOF-808-NaCu before and after catalysis.

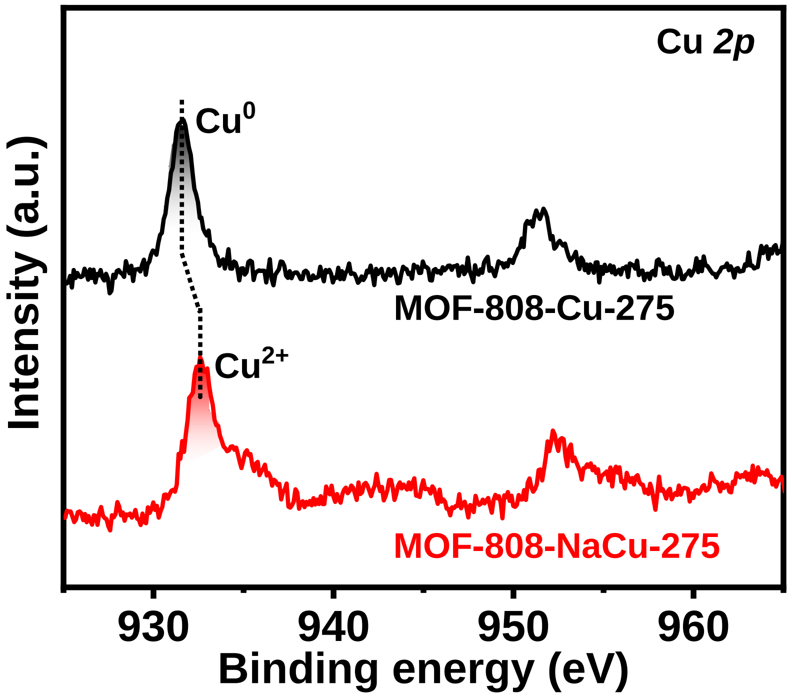

Figure S18. The Cu *2p* XPS spectra of MOF-808-NaCu and MOF-808-Cu after the hydrogenation reaction at 275 ℃. Typically, Cu^{2+} can be preserved in MOF-808-NaCu (with a binding energy of 932.9 eV) but reduced to Cu^{0} in MOF-808-Cu (with a binding energy of 931.6 eV) after the reaction.

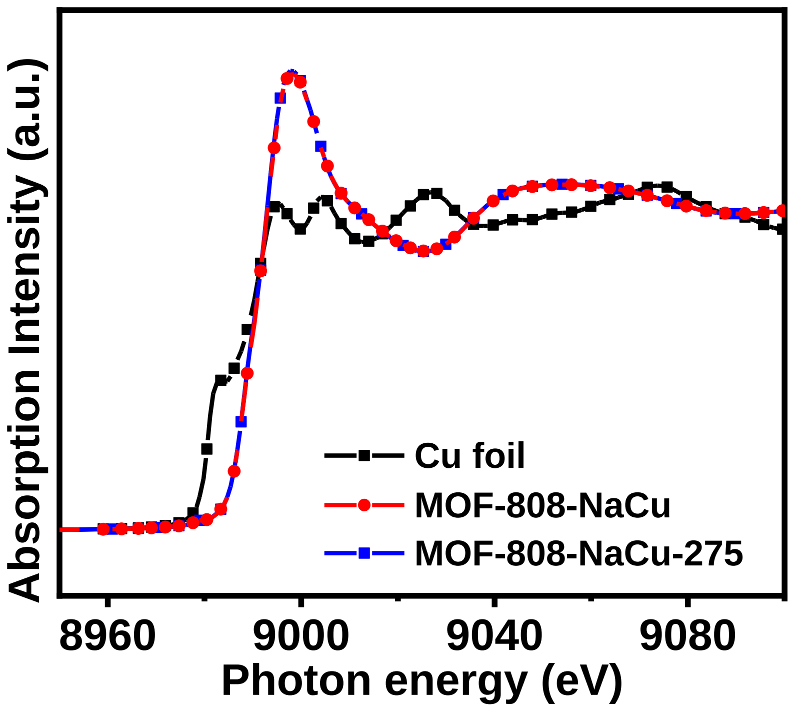

Figure S19. The Cu K-edge XANES spectra of MOF-808-NaCu after the hydrogenation reaction at 275 ℃. The XANES spectrum of MOF-808-NaCu after catalysis is almost identical to that of as-synthesized MOF-808-NaCu, implying that the structure of the single-atom Cu sites is preserved after catalysis.

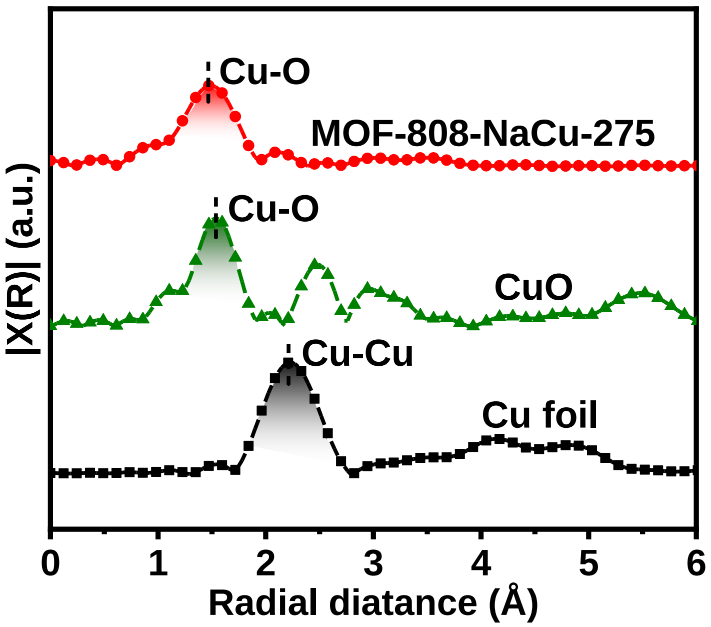

Figure S20. The FT-EXAFS spectra of MOF-808-NaCu after the CO_2 hydrogenation reaction at 275 ℃ and the reference samples. The Cu-O scattering can be observed at approximately 1.52 Å without the Cu-Cu scattering, verifying the preserved atomic dispersity of Cu species after catalysis.

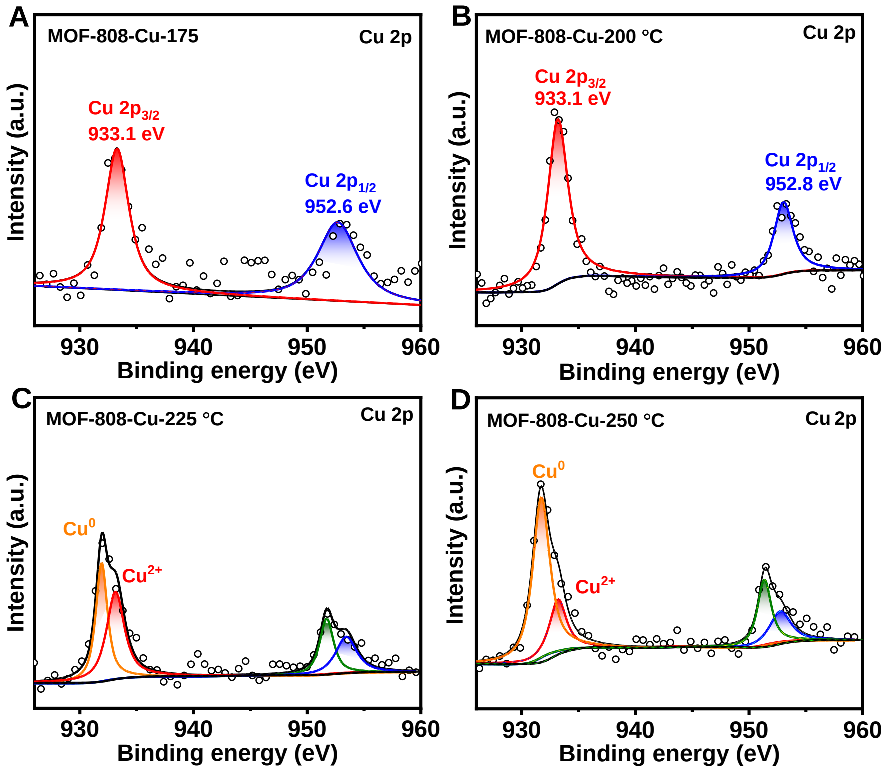

Figure S21. The Cu 2p XPS spectra of MOF-808-Cu after the hydrogenation reaction at (*A*) 175 ℃, (*B*) 200 ℃, (*C*) 225 ℃ and (*D*) 250 ℃. Typically, Cu^{2+} can be retained after reaction ≤200 °C, while the Cu^0 species emerges as the temperature further increases.

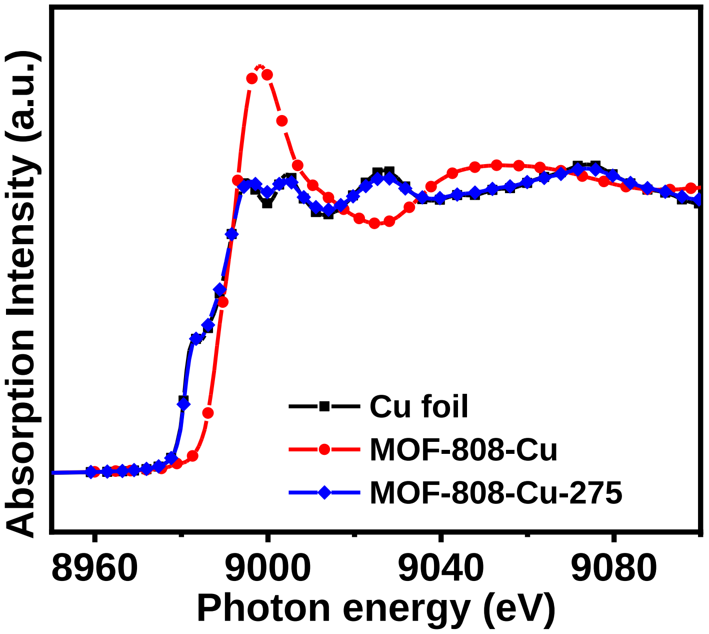

Figure S22. The Cu K-edge XANES spectrum of MOF-808-Cu after the CO_2 hydrogenation reaction at 275 ℃, which is very different from that of as-syntheszed MOF-808-Cu but similar to that of Cu foil, reflecting the sintering of single-atom Cu sites after catalysis.

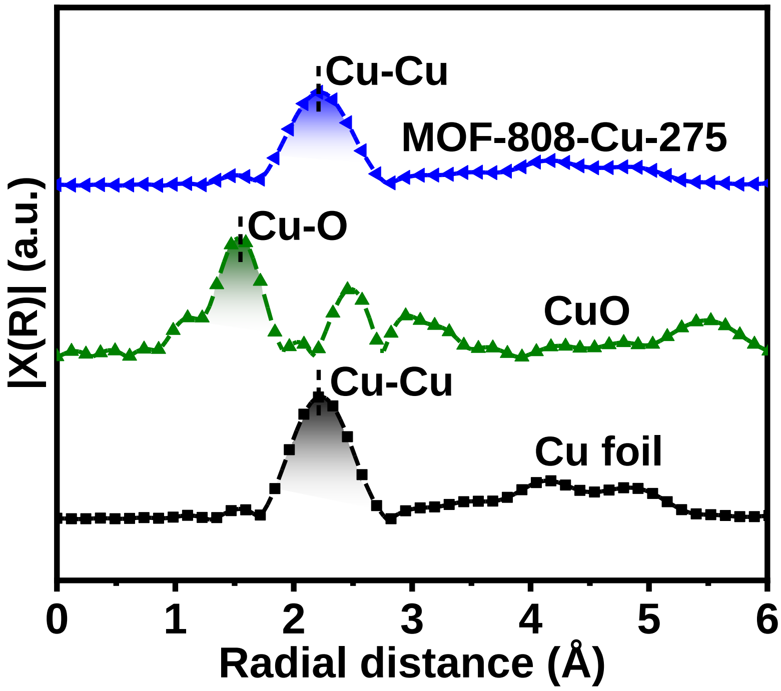

Figure S23. The FT-EXAFS spectra of MOF-808-Cu after the CO_2 hydrogenation reaction at 275 ℃ and the reference samples. The Cu-Cu scattering can be observed at approximately 2.21 Å, implying sintering during catalysis.

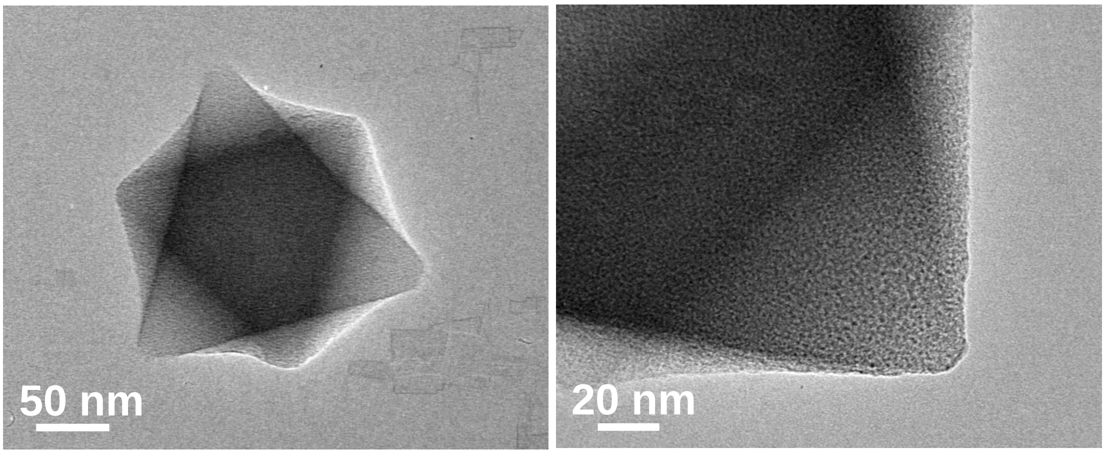

Figure S24. TEM images of MOF-808-NaCu after the CO_2 hydrogenation reaction at 275 ℃. No nanoparticles can be observed.

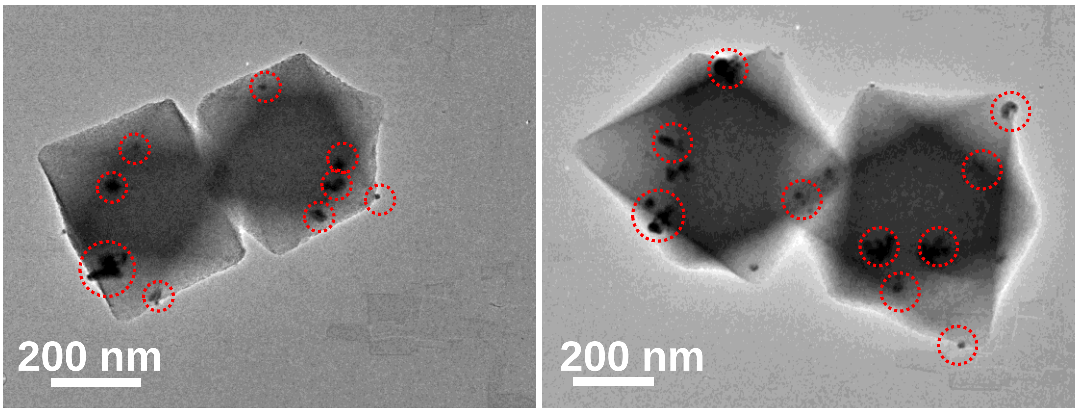

Figure S25. TEM images of MOF-808-Cu after the CO_2 hydrogenation reaction at 275 ℃. Nanoparticles are highlighted by red circles.

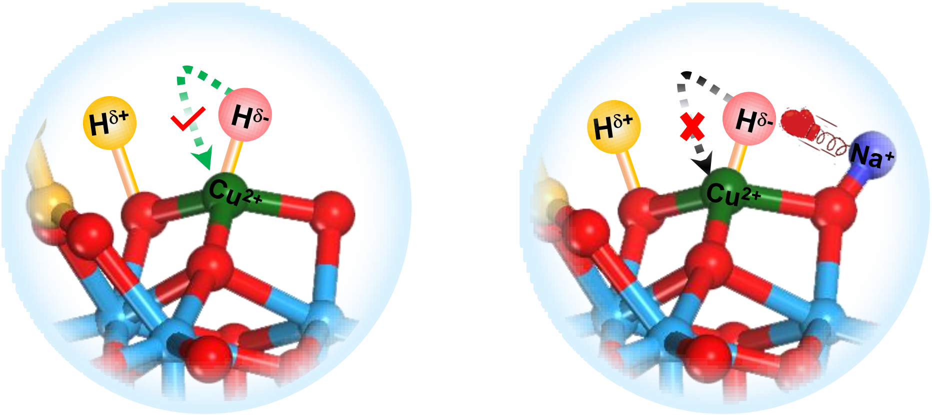

Figure S26. Schematic representation of electrostatic interaction between Na^+ (or $H^{\delta+}$) with the adjacent $H^{\delta-}$ within MOF-808-NaCu (right), compared to MOF-808-Cu (left), illustrating how the reductive elimination process is suppressed in MOF-808-NaCu.

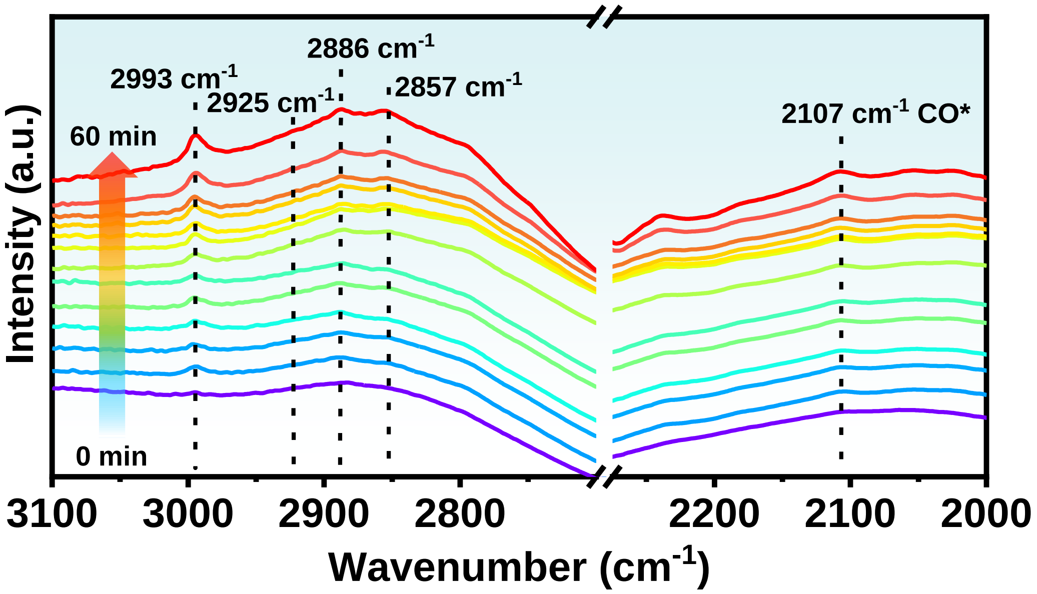

Figure S27. *In-situ* DRIFT spectra of the CO_2/H_2 reaction on MOF-808-Cu (test conditions: CO_2/H_2 volume ratio of 1/3, flow rate of 16 mL min^{-1}, 225 ℃). The CO* peak (2107 cm^{-1}) is readily detectable upon introducing the reaction mixture, and the other intermediates are formed along with the generation of CO*, such as HCOO* (2993 and 2886 cm^{-1}) and H_2CO* (2925 and 2857 cm^{-1}), indicating the coexistence of two pathways.

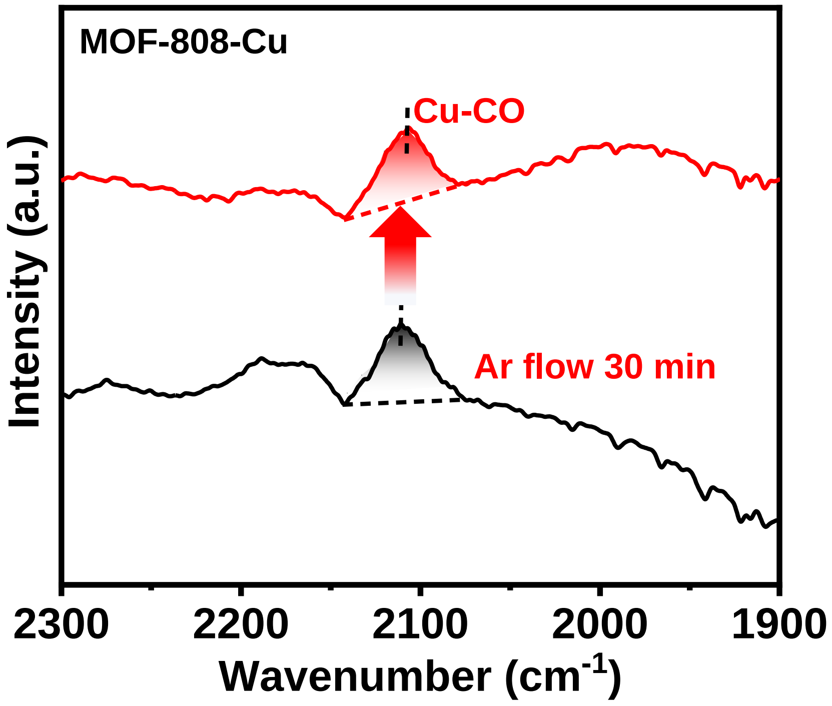

Figure S28. *In-situ* CO-DRIFTS spectra over MOF-808-Cu. The CO adsorption peak remains stable after Ar purging for 30 min.

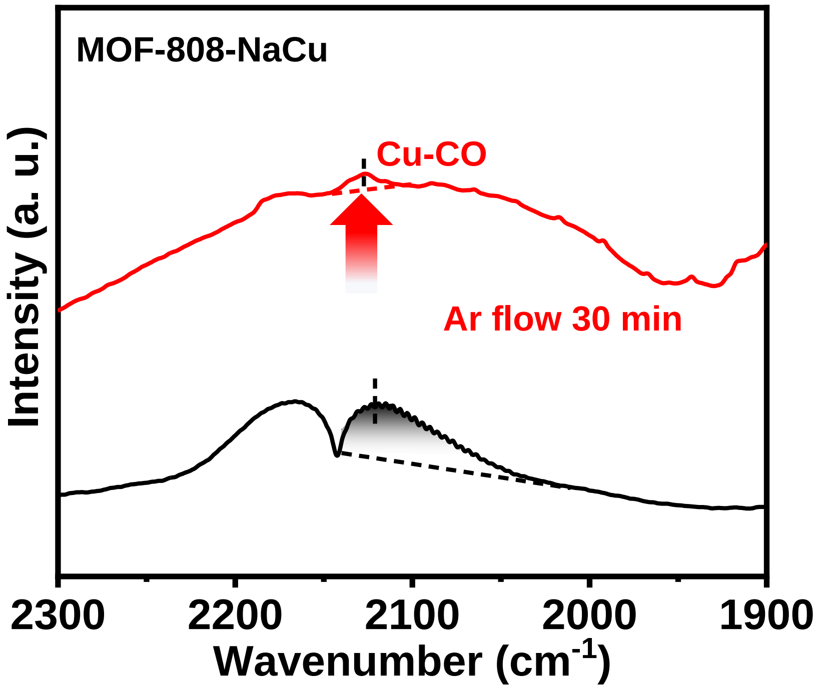

Figure S29. *In-situ* CO-DRIFTS spectra of MOF-808-NaCu. The CO adsorption peak almost disappears after Ar purging for 30 min.

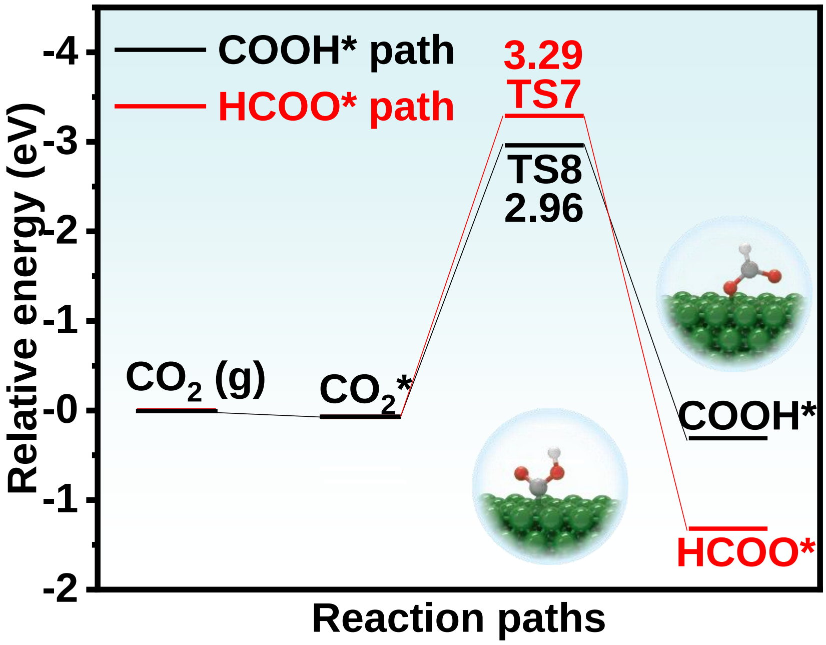

Figure S30. Energy profiles of CO_2 hydrogenation paths on Cu NPs (inset, the structures of intermediate geometries, green, Cu; red, O; white, H; C of CO_2, grey).

Table S1. The loading amounts of Cu and Na in the as-prepared samples.

Samples	Na content (wt%)[a]	Cu content (wt%)[a]	Molar ratio[b]
MOF-808-Na	5.3	-	Zr : Na = 6 : 3.6
MOF-808-Cu	-	4.1	Zr : Cu = 6 : 0.9
MOF-808-NaCu	3.2	4.5	Zr : Na : Cu = 6 : 2 : 1

[a] The contents of Na/Cu are determined by ICP–OES;

[b] The Zr content is determined by the residual weight after deducting Cu/Na amount (decided by ICP) and the repeating structure unit in MOF-808, $[Zr_6(\mu_3\text{-}O)_4(\mu_3\text{-}OH)_4(HCO_2)_6(BTC)_2]$.

References

1. Liu H, Xu C and Li D *et al.* Photocatalytic Hydrogen Production Coupled with Selective Benzylamine Oxidation over MOF Composites. *Angew Chem Int Ed* 2018; **57**: 5379-83.
2. Perdew J P, Burke K and Ernzerhof M. Generalized gradient approximation made simple *Phys Rev Lett* 1996; **7**: 3865. 10.1103/PhysRevLett.77.3865.
3. Kresse G and Joubert D. From ultrasoft pseudopotentials to the projector augmented-wave method. *Phys Rev B* 1999; **59**: 1758.
4. Kresse G and Furthmüller J. Efficiency of ab-initio total energy calculations for metals and semiconductors using a plane-wave basis set. *Comp Mat Sci* 1996; **6**: 15-50.
5. Grimme S, Antony J and Ehrlich S *et al.* A consistent and accurate ab initio parametrization of density functional dispersion correction (DFT-D) for the 94 elements H-Pu. *J Chem Phys* 2010; **132**: 154104.
6. Henkelman G, Uberuaga B P and Jónsson H A. climbing image nudged elastic band method for finding saddle points and minimum energy paths. *J Chem Phys* 2000; **113**: 9901-4.
7. Xue W, Song X and Mei D. Theoretical insights into CO oxidation over MOF-808 encapsulated single-atom metal catalysts. *J Phys Chem C* 2021; **31**: 17097.
8. Xu X, Zhang Z and Dong J *et al.* Ultrafast epitaxial growth of metre-sized single-crystal graphene on industrial Cu foil. Sci Bull 2017; **62**: 1074-80.
